# Bacterial outer membrane vesicle-cancer cell hybrid membrane-coated nanoparticles for sonodynamic therapy in the treatment of breast cancer bone metastasis

**DOI:** 10.1186/s12951-024-02619-w

**Published:** 2024-06-10

**Authors:** Jiahao Wang, Shuailong Liang, Sijie Chen, Tianliang Ma, Mingyu Chen, Chengcheng Niu, Yi Leng, Long Wang

**Affiliations:** 1 Department of Orthopedics, Xiangya Hospital, Central South University, Changsha, China; 2 The School of Medicine, Nankai University, Tianjin, 300071 China; 3 Hunan Engineering Research Center of Biomedical Metal and Ceramic Implants, Xiangya Hospital, Central South University, Changsha, China; 4 National Clinical Research Center for Geriatric Disorders, Xiangya Hospital, Central South University, Changsha, China; 5 Department of Ultrasound Diagnosis, Second Xiangya Hospital, Central South University, Changsha, China; 6 Department of Rehabilitation, Xiangya Hospital, Central South University, Changsha, China; 7 Hunan Key Laboratary of Aging Biology, Xiangya Hospital, Central South University, 87 Xiangya Road, Kaifu District, Changsha, Hunan 410008 China

**Keywords:** Breast cancer bone metastasis, Sonodynamic therapy, Bacterial outer membrane vesicle, Hybrid membrane, Biomimetic nanoparticles

## Abstract

Breast cancer bone metastasis is a terminal-stage disease and is typically treated with radiotherapy and chemotherapy, which causes severe side effects and limited effectiveness. To improve this, Sonodynamic therapy may be a more safe and effective approach in the future. Bacterial outer membrane vesicles (OMV) have excellent immune-regulating properties, including modulating macrophage polarization, promoting DC cell maturation, and enhancing anti-tumor effects. Combining OMV with Sonodynamic therapy can result in synergetic anti-tumor effects. Therefore, we constructed multifunctional nanoparticles for treating breast cancer bone metastasis. We fused breast cancer cell membranes and bacterial outer membrane vesicles to form a hybrid membrane (HM) and then encapsulated IR780-loaded PLGA with HM to produce the nanoparticles, IR780@PLGA@HM, which had tumor targeting, immune regulating, and Sonodynamic abilities. Experiments showed that the IR780@PLGA@HM nanoparticles had good biocompatibility, effectively targeted to 4T1 tumors, promoted macrophage type I polarization and DC cells activation, strengthened anti-tumor inflammatory factors expression, and presented the ability to effectively kill tumors both in vitro and in vivo, which showed a promising therapeutic effect on breast cancer bone metastasis. Therefore, the nanoparticles we constructed provided a new strategy for effectively treating breast cancer bone metastasis.

**Graphical Abstract:**

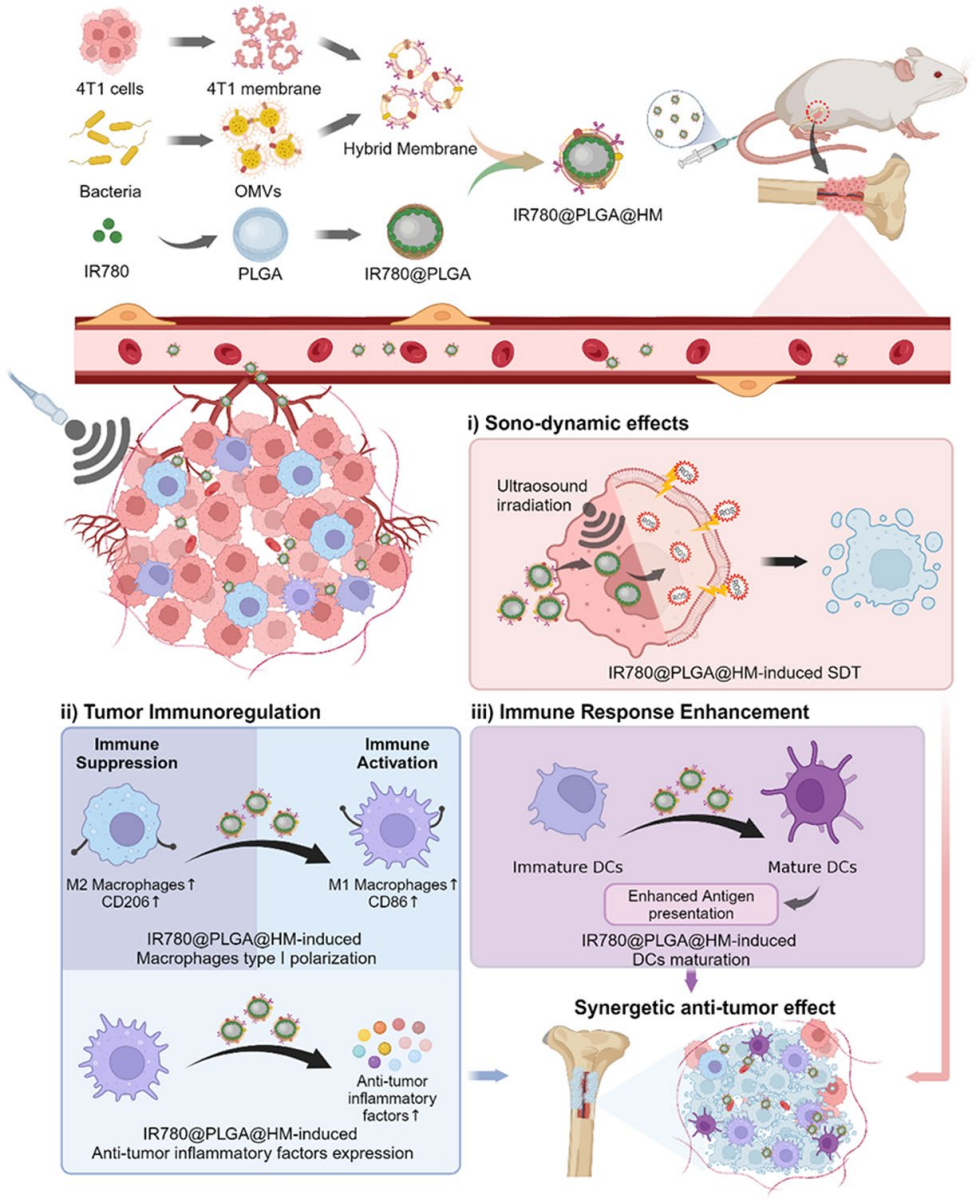

**Supplementary Information:**

The online version contains supplementary material available at 10.1186/s12951-024-02619-w.

## Introduction

Breast cancer is the most common malignant tumor in women, with approximately 2.3 million new cases worldwide in 2020, making it the leading type of cancer [1]. Skeletal sites are the most common sites for metastasis in breast cancer, accounting for 60–75% of all metastatic breast cancer cases [2]. Approximately 5% of breast cancer patients experience bone metastasis at the time of initial diagnosis, and 50% of advanced breast cancer patients have bone metastasis [3, 4]. According to the American Cancer Society (ACS), the 5-year survival rate for breast cancer patients is 91%, and the 10-year survival rate is 84% [5]. However, for patients with bone metastasis, the 3-year survival rate is only 50.5%, with a median survival period of 36 months [6]. Bone metastasis is often associated with skeletal-related events (SREs) such as bone pain, pathological fractures, spinal cord compression, and hypercalcemia, significantly impacting patients’ autonomy and quality of life [7]. Currently, besides foundational treatments like endocrine therapy and chemotherapy, targeted therapies and immunotherapeutic drugs, such as PARP inhibitors, and immune checkpoint inhibitors (PD-1, PD-L1, and CTLA4), have shown efficacy in inhibiting tumor progression [8]. However, the development of new and more effective treatment approaches is still underway.

In recent years, Sonodynamic Therapy (SDT) has emerged as an innovative and promising approach in the field of cancer treatment. This therapeutic method involves the administration of a sonosensitizer to the target tissue, followed by the application of ultrasound waves to activate the sonosensitizer, thereby generating reactive oxygen species (ROS) within the tissue and selectively eliminating targeted tumor cells. Among the various sonosensitizers, IR780 iodide stands out as an exceptional ultrasound-responsive agent. It exhibits a peak optical absorption at 780 nm wavelength, robust fluorescence intensity, and favorable biosafety, making it an ideal candidate for cancer treatment. Previous studies have substantiated its efficacy in inducing cancer cell death through the generation of ROS [9–12]. Additionally, the “nano ghost” strategy, utilizing cell membrane-coated biomimetic nanoparticles, enhances nanoparticle delivery to carcinomas, leading to elevated local drug concentrations and heightened tumor-destructive effects [13]. This coating includes membranes from various sources, such as red blood cells, platelets, immune cells, and cancer cells [14–17]. Notably, the cancer cell membrane demonstrates the capability to guide nanoparticles toward homotypic tumors, leveraging self-adherence effects among cancer cells. This phenomenon is attributed to interactions involving Thomsen-Friedenreich antigens and E-cadherin on the cell surface [18]. The coating with cancer cell membrane has exhibited enhanced efficacy in nanodrug infiltration, showcasing promising outcomes in diverse cancer treatment studies and presenting impactful strategies for tumor nano therapy [19–21].

Bacteria-derived patterns have been studied to apply as immune regulators for cancer treatment [22–24]. Bacterial outer membrane vesicles (OMVs), with nano-sized lipid-bilayer vesicular structures and immunostimulatory components, are secreted by Gram-negative bacteria and possess the ability to regulate tumor microenvironment, potentiating antitumor response for immunotherapy. In previous studies, researchers utilized OMVs as cancer vaccines [25], anti-tumor drugs [26–28], or drug delivery systems [29, 30], all of which have witnessed their immune regulation and tumor inhibition effect during treatment. Kim et al. reported the tumor inhibition effect of systematically administrated OMVs, through an interferon-γ dependent pathway [26]. Zou et al. found that hybrid vesicles based on OMVs could promote the activation of DC cells and T lymphocytes, furthermore, inhibiting the lung metastasis of 4T1 tumors [28]. Combining OMVs with other cancer therapies is supposed to enhance the whole antitumor effect, which has been little investigated yet.

In this study, we designed and fabricated a novel hybrid membrane (HM) by integrating membranes from breast cancer cells (4T1) and OMVs obtained from E. coli DH5α. We employed this HM to coat poly (lactic-co-glycolic acid) (PLGA) nanoparticles encapsulating IR780 (referred to as IR780@PLGA@HM). The purpose behind this innovative construct was to synergistically harness the potential of SDT and amplify immunotherapeutic responses for effectively addressing breast cancer bone metastasis.

## Results and discussion

**Fig. 1:**
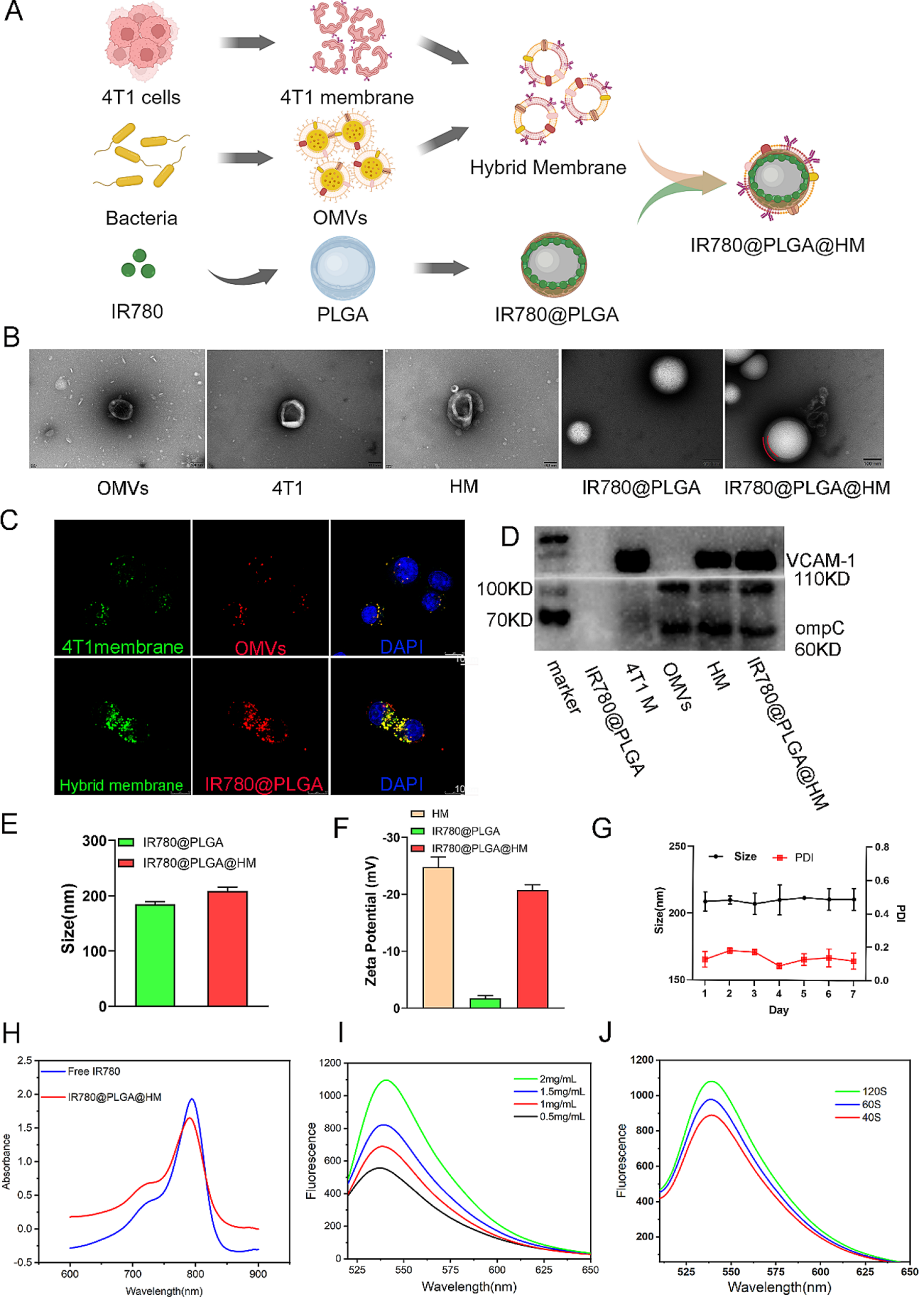
Characterization of IR780@PLGA@HM nanoparticles. (**A**) Schematic illustration of IR780@PLGA@HM nanoparticles construction. (**B**) TEM observation of OMVs, 4T1 membrane, HM, IR780@PLGA, and IR780@PLGA@HM (Scale bar = 100 nm. Red lines marked HM coating on nanoparticles). (**C**) CLSM images of 4T1 cells’ uptake of HM (DiO labeled 4T1 membrane as green and DiI labeled OMVs as red) or IR780@PLGA@HM nanoparticles (DiO labeled HM as green and DiI labeled IR780@PLGA as red) (Scale bar = 10 μm). (**D**) Western blot verification of HM and IR780@PLGA@HM nanoparticles. (**E**) Size and (**F**) Zeta potential of IR780@PLGA and IR780@PLGA@HM nanoparticles. (**G**) Size and PDI of IR780@PLGA@HM nanoparticles in PBS at 4℃ for 7 days. (**H**) UV-vis-NIR absorption spectra of free IR780 and IR780@PLGA@HM nanoparticles. (**I**) Concentration-dependent and (**J**) Time-dependent ROS generation of IR780@PLGA@HM nanoparticles with US irradiation (2 W/cm^2^, 1 MHz), with SOSG as fluorescence probe

In this research, we constructed IR780@PLGA@HM nanoparticles for SDT and immune regulation to strengthen the therapeutic efficacy of Breast Cancer Bone Metastasis. We used a single emulsion evaporation method to prepare nanoparticles [31], constructed a hybrid membrane, and coated them onto IR780@PLGA as previously reported [28, 32](Fig. 1A). The TEM images showed that OMVs, 4T1 membrane, and HM all had regular vesicle structures, while IR780@PLGA represented a uniform spherical shape. After coating, IR780@PLGA@HM particles revealed the expected spherical shape and core-shell structures with HM as the outer membrane, marked by red lines. The diameters of them were all around 200 nm (Fig. 1B). We labeled membranes and nanoparticles with fluorescence dye and observed them under CLSM to verify whether membrane infusion and coating were successful. We used DiO to label 4T1 membrane with green and DiI to label OMVs with red fluorescence. After 4T1 cells’ uptake of the hybrid membrane, we observed both fluorescence overlapped in the merged image, showing that 4T1 membrane was successfully infused with OMVs.

Further, we labeled HM with DiO and IR780@PLGA with DiI, finding that most of the green and red fluorescence colocalized within 4T1 cells. The overlapped fluorescence revealed that HM had been coated on IR780@PLGA nanoparticles (Fig. 1C). To verify the contents of nanoparticles, we utilized Western blot to detect the 4T1 cell-specific protein, VCAM-1, and bacteria-specific marker, ompC in nanoparticles. IR780@PLGA didn’t show any protein components. HM contained both VCAM-1 expressed on 4T1 membrane and ompC on bacteria OMVs. After coating HM onto IR780@PLGA, IR780@PLGA@HM particles also obtained the protein signature (Fig. 1D). The average hydrodynamic sizes of IR780@PLGA and IR780@PLGA@HM nanoparticles were 180–190 nm and 200–215 nm, respectively, revealed by DLS (Fig. 1E). The Zeta Potential of HM and IR780@PLGA were all negative, while the Potential of IR780@PLGA@HM was in the range of HM and IR780@PLGA (Fig. 1F). When suspended in PBS at 4℃ for 7 days, IR780@PLGA@HM revealed little changes in the size and PDI (lower than 0.2) measured by DLS (Fig. 1G), showing that IR780@PLGA@HM were stable enough to work for in vivo study. UV-vis-NIR absorption spectra verified the IR780 component in IR780@PLGA@HM and showed that both IR780 and nanoparticles had a characteristic absorption peak at 780 nm, indicating that IR780@PLGA@HM had loaded the sonodynamic agent successfully (Fig. 1H). IR780@PLGA particles had an encapsulation efficiency of 29.59% and a drug-loading efficiency of 0.32%, which was calculated via a standard curve of IR780 (Figure S1). Furthermore, to confirm the sonodynamic ability of IR780@PLGA@HM, we applied SOSG to detect ROS generation through fluorescence intensity in vitro. When exposed to the same ultrasound irradiation (2 W/cm^2^, 1 MHz) for 30 s, IR780@PLGA@HM nanoparticles presented dose-dependently increasing production of ROS (Fig. 1I). The fluorescence intensity also increased time-dependently when nanoparticles (1.5 mg/mL) were treated with prolonged irradiation time from 40 s to 120 s (Fig. 1J). Therefore, IR780@PLGA@HM nanoparticles responded well to ultrasound after HM coating and could serve as a potential sonodynamic therapy.

**Fig. 2:**
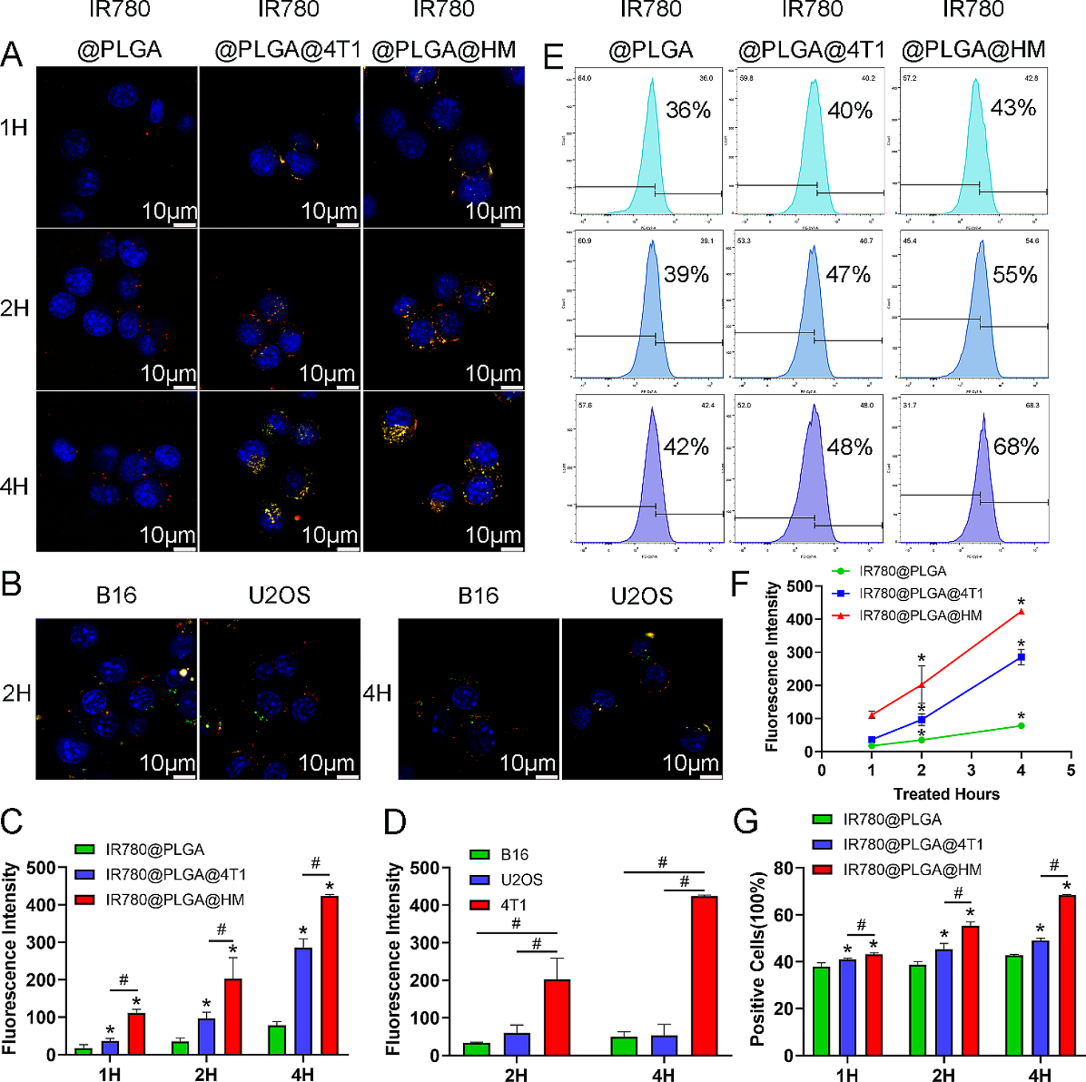
Evaluation of homotypic-targeting capability of IR780@PLGA@HM nanoparticles in vitro. (**A**) 4T1 cells, (**B**) B16 cells, and U2OS cells’ uptake of nanoparticles were imaged by CLSM (DiO labeled HM as green and DiI labeled IR780@PLGA as red; Scale bar = 10 μm) and quantified in (**C**) and (**D**). Changes in fluorescence intensity of nanoparticles in (A) were quantified in (**F**). (**E**) Flow cytometry detected IR780 positive 4T1 cells treated with nanoparticles, and quantification results were in (**G**). Statistical significances were calculated via Student’s t-test, *p < 0.05 (IR780@PLGA as the control group). ^#^*p* < 0.05 (difference between compared groups)

After successful construction, we evaluated the homotypic-targeting capability of IR780@PLGA@HM in vitro by using 4T1 cells, B16 cells, and U2OS cells. We labeled 4T1 membrane or HM as green with DiO and PLGA as red with DiI when constructing IR780@PLGA@HM nanoparticles. Therefore, we could visualize the cells’ uptake of nanoparticles under CLSM after culturing nanoparticles with 4T1 cells for 1 h, 2 h, and 4 h. We found that more and more nanoparticles were taken up as time went by (Fig. 2A). 4T1 membrane and HM-coated nanoparticles got more into 4T1 cells compared with naked IR780@PLGA nanoparticles. Then the red fluorescence intensity of PLGA was quantified to show the amounts of nanoparticles getting into 4T1 cells (Fig. 2C). At all three time points, IR780@PLGA@4T1 and IR780@PLGA@HM nanoparticles were taken up significantly more than IR780@PLGA. HM coating presented an even better targeting ability than 4T1 membrane. Besides, we detected the uptake efficiency of IR780@PLGA@HM between RAW264.7 cells and 4T1 cells, as shown in Figure S2. Four hours after the administration of nanoparticles, we found that IR780@PLGA@HM was taken into 4T1 cells more than RAW264.7 cells, indicating that the targeting-promoting effect of HM mainly contributed to the homologous ability of 4T1 membrane, although OMV components possibly promote the endocytosis of nanoparticles by immune cells. After that, we cultured IR780@PLGA@HM nanoparticles with another two different cancer cells, B16 cells, and U2OS cells, to verify the 4T1 specific targeting ability of HM coating. After incubating for 2 h and 4 h, 4T1 cells’ uptake was significantly more than B16 and U2OS cells, while there was no significant difference between these two cells (Fig. 2B, D), indicating that HM coating nanoparticles obtained the homotypic-targeting capability of 4T1 cells membrane. Flow cytometry observed similar results. IR780 positive 4T1 cells increased from 36 to 42%, 40–48%, and 43–68% after being treated with IR780@PLGA, IR780@PLGA@4T1, and IR780@PLGA@HM nanoparticles, respectively, from 1 h to 4 h (Fig. 2E). Quantification results showed that IR780 fluorescence intensity within 4T1 cells increased time-dependently in three groups (Fig. 2F). There were more IR780 positive cells in IR780@PLGA@4T1, and IR780@PLGA@HM groups compared with the naked IR780@PLGA group. IR780@PLGA@HM treatment also induced more positive 4T1 cells than IR780@PLGA@4T1 group (Fig. 2G), indicating that OMV further promoted 4T1 cells’ uptake of nanoparticles and enhanced the targeting ability of IR780@PLGA@HM nanoparticles.

**Fig. 3:**
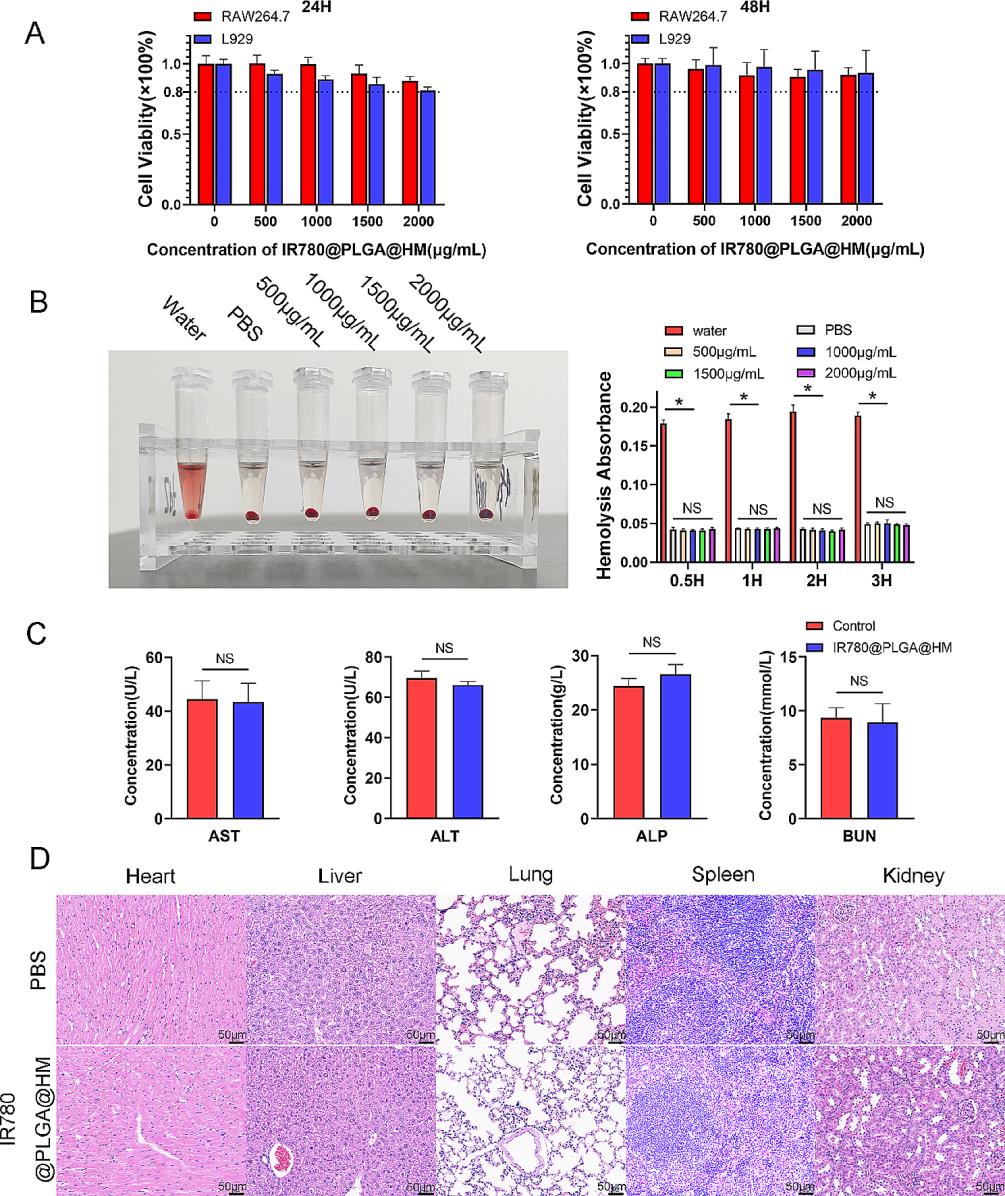
Evaluation of biosafety of IR780@PLGA@HM nanoparticles. (**A**) Cell viability of RAW264.7 cells and L929 cells were evaluated after 24 h and 48 h of treatment with IR780@PLGA@HM nanoparticles at different concentrations. (**B**) Hemolysis observation of IR780@PLGA@HM nanoparticles at different concentrations and quantification results. (**C**) Blood biochemistry analysis of liver and kidney function indicators: AST, ALT, ALP, BUN, and (**D**) HE staining of major organs from BALB/C mice, after intravenous injection of IR780@PLGA@HM nanoparticles (Scale bar = 50 μm). Statistical significances were calculated via Student’s t-test, *p < 0.05. NS meant no significant difference

We used RAW264.7 cells and L929 cells to detect the influence of IR780@PLGA@HM nanoparticles on cell viability in vitro, revealing that more than 80% of cells survived well after 24-hour and 48-hour treatment of IR780@PLGA@HM nanoparticles with concentrations ranging from 0 to 2000 µg/mL (Fig. 3A). Besides, we evaluated the sonodynamic cytotoxicity of IR780@PLGA@HM against RAW264.7 and L929 cells. As shown in Figure S4, both cells presented a dose-dependent decrease in cell viability after the administration of nanoparticles under ultrasound irradiation (1 W/cm2, 1 MHz, 10 s on and 10 s off for 2 min). However, more than 70% of L929 cells and 60% of RAW264.7 cells were still alive in 2000 µg/mL nanoparticles treated groups, which indicated that IR780@PLGA@HM had good biosafety. Fresh red blood cells were treated with IR780@PLGA@HM nanoparticles for 3 h. Water and PBS-treated groups were regarded as the positive and negative control groups, respectively. Results showed that nanoparticles had no impact on red blood cell integrity, with no significant difference in released heme between the nanoparticles groups and PBS group (Fig. 3B). These results indicated that IR780@PLGA@HM nanoparticles had little cytotoxicity in vitro. Continuingly, we injected nanoparticles intravenously into BALB/C mice to evaluate the in vivo biosafety of nanoparticles. After treating mice for 2 weeks, AST, ALT, ALP, BUN (Fig. 3C), UA, and TP (Figure S3) all didn’t show significant differences between PBS and nanoparticle-treated groups. IR780@PLGA@HM didn’t make any changes in blood cell counts (Figure S3), either. HE staining confirmed that IR780@PLGA@HM caused no obvious changes to the morphology of the main organs, including the heart, liver, lung, spleen, and kidney (Fig. 3D). Therefore, IR780@PLGA@HM nanoparticles also had good biosafety in vivo. It could be used for in vivo treatment.

**Fig. 4:**
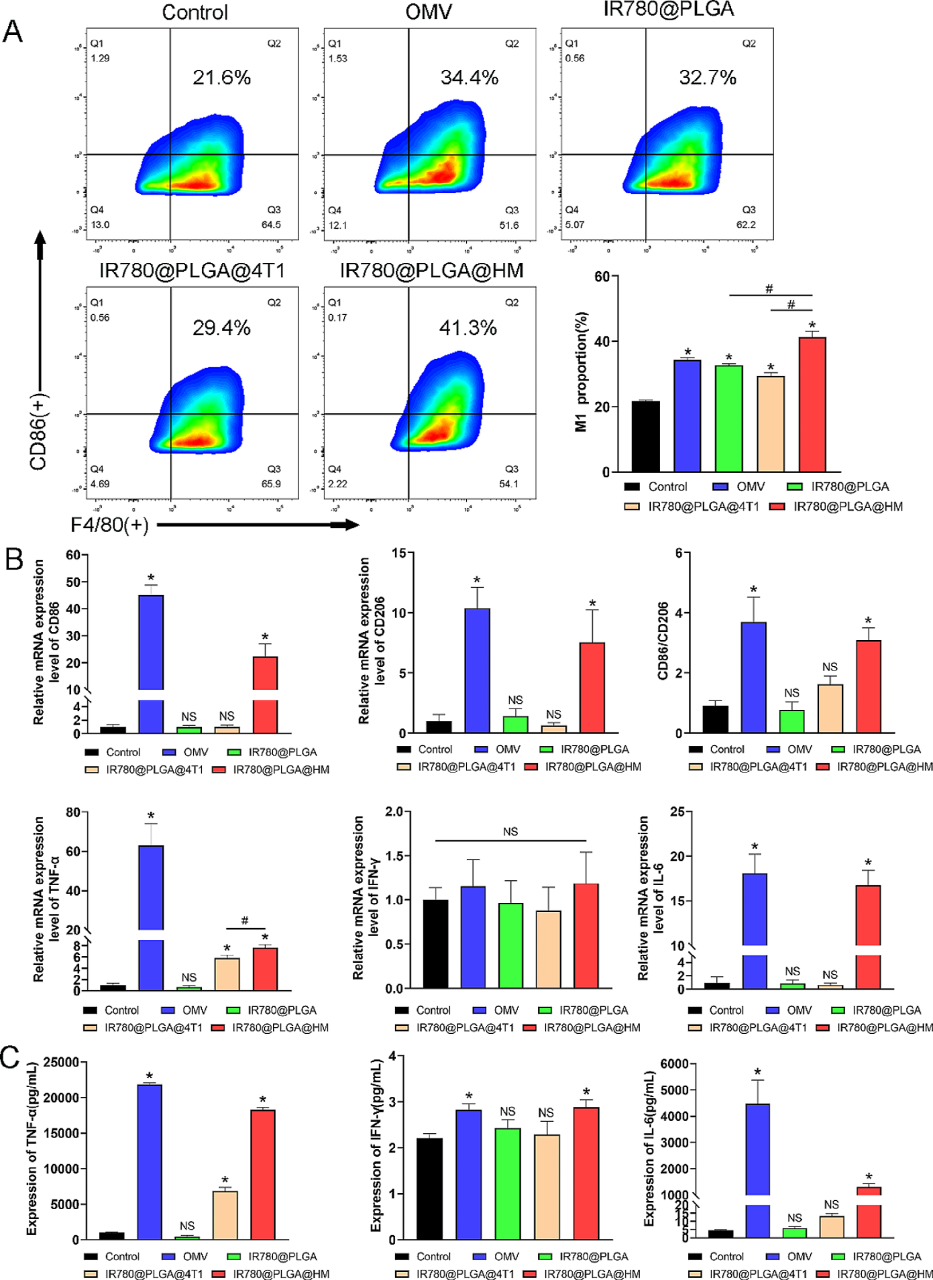
Evaluation of macrophage polarization in vitro. (**A**) Flowcytometry showed regulation of nanoparticles on RAW264.7 cells polarization in vitro, with quantification results. (**B**) qRT-PCR showed macrophages polarization and activation relevant gene expression (CD86, CD206, CD86/CD206, TNF-α, IFN-γ and IL-6). (**C**) CBA detected inflammatory cytokines expression (TNF-α, IFN-γ, and IL-6) of RAW264.7 cells after nanoparticle treatment. Statistical significances were calculated via Student’s t and One-way ANOVA test, *p < 0.05 (compared with the control group). ^#^*p* < 0.05 (difference between compared groups). NS meant no significant difference

According to previous research, OMV could stimulate the immune response, promote macrophage activation, and inhibit tumor growth by increasing IFN-γ expression [28, 33, 34], therefore, it needed to detect whether IR780@PLGA@HM obtained the ability of OMVs to regulate the immune response. Flow cytometry revealed that OMV could significantly promote RAW264.7 cells M1 polarization, from 21.6 to 34.4% (Fig. 4A). IR780@PLGA and IR780@PLGA@4T1 nanoparticles could also increase M1 proportion percent to 29.4% and 32.7%, respectively, lower than OMV treatment. IR780@PLGA@HM nanoparticles induced 41.3% M1 polarization, the most among all groups, indicating that infusing OMV successfully improved the macrophage activation effect of IR780@PLGA@HM nanoparticles. Besides, we separated the primary murine bone marrow-derived macrophage cells and treated them with different nanoparticles. As shown in Figure S5, IR780@PLGA@HM could also promote BMDM M1 polarization, from 2.3 to 66.1%. OMV significantly promoted M1 polarization to 56.7%. IR780@PLGA and IR780@PLGA@4T1 raised the M1 proportion to 27.2% and 22.6%, respectively. We presented that IR780@PLGA could promote both RAW264.7 and BMDM M1 polarization. Although PLGA has shown good biosafety in this research and previous studies, limited research has reported their direct influence on macrophage polarization before. One research investigated the influence of intravenously administrated PLGA nanoparticles on the development of aortic atherosclerotic plaques. They found that injection of PLGA for 4 to 12 weeks significantly increased the extension of atherosclerotic plaques and the expression of associated inflammatory factors, such as TNF-α and IL-6, indicating the potential pro-inflammation effect of PLGA nanoparticles [35]. However, further investigations are required to explore the effect of PLGA on macrophage polarization and evaluate the influence of PLGA on organisms in vivo from more aspects. Quantitative Real-time PCR detected gene expression related to macrophage polarization. CD86 expression was significantly upregulated by OMVs and IR780@PLGA@HM nanoparticles, while IR780@PLGA and IR780@PLGA@4T1 nanoparticles made no significant changes to CD86 gene expression (Fig. 4B). Although CD206 expression was also elevated by OMV and IR780@PLGA@HM, the CD86/CD206 ratio of OMV and IR780@PLGA@HM nanoparticles groups was significantly more than the control group, demonstrating that RAW264.7 cells polarized mostly into type I with the administration of OMV and IR780@PLGA@HM nanoparticles (Fig. 4B). Besides, TNF-α and IL-6 gene expression of macrophages were raised in these two groups as well, while OMV induced more gene expression than IR780@PLGA@HM nanoparticles. IR780@PLGA@4T1 nanoparticles also promoted TNF-α expression, which was significantly lower than IR780@PLGA@HM. No significant difference was found in IFN-γ expression among groups, however, OMV and IR780@PLGA@HM nanoparticles groups showed relatively higher IFN-γ expression levels than other groups (Fig. 4B). Further, we detected cytokines secretion in culture supernatants of macrophages. RAW264.7 cells secreted significantly more TNF-α, IFN-γ, and IL-6 after OMV and IR780@PLGA@HM nanoparticles treatment than the control group (Fig. 4C). IR780@PLGA@4T1 could also promote TNF-α secretion, consistent with the upregulation of TNF-α gene expression, nevertheless, it is not as much as OMV and IR780@PLGA@HM nanoparticles. Considering all these results, we found that OMV infusion endowed IR780@PLGA@HM nanoparticles with the ability to activate macrophage type I polarization and enhance pro-inflammation cytokines expression, which had a proven role in anti-tumor therapy.

**Fig. 5:**
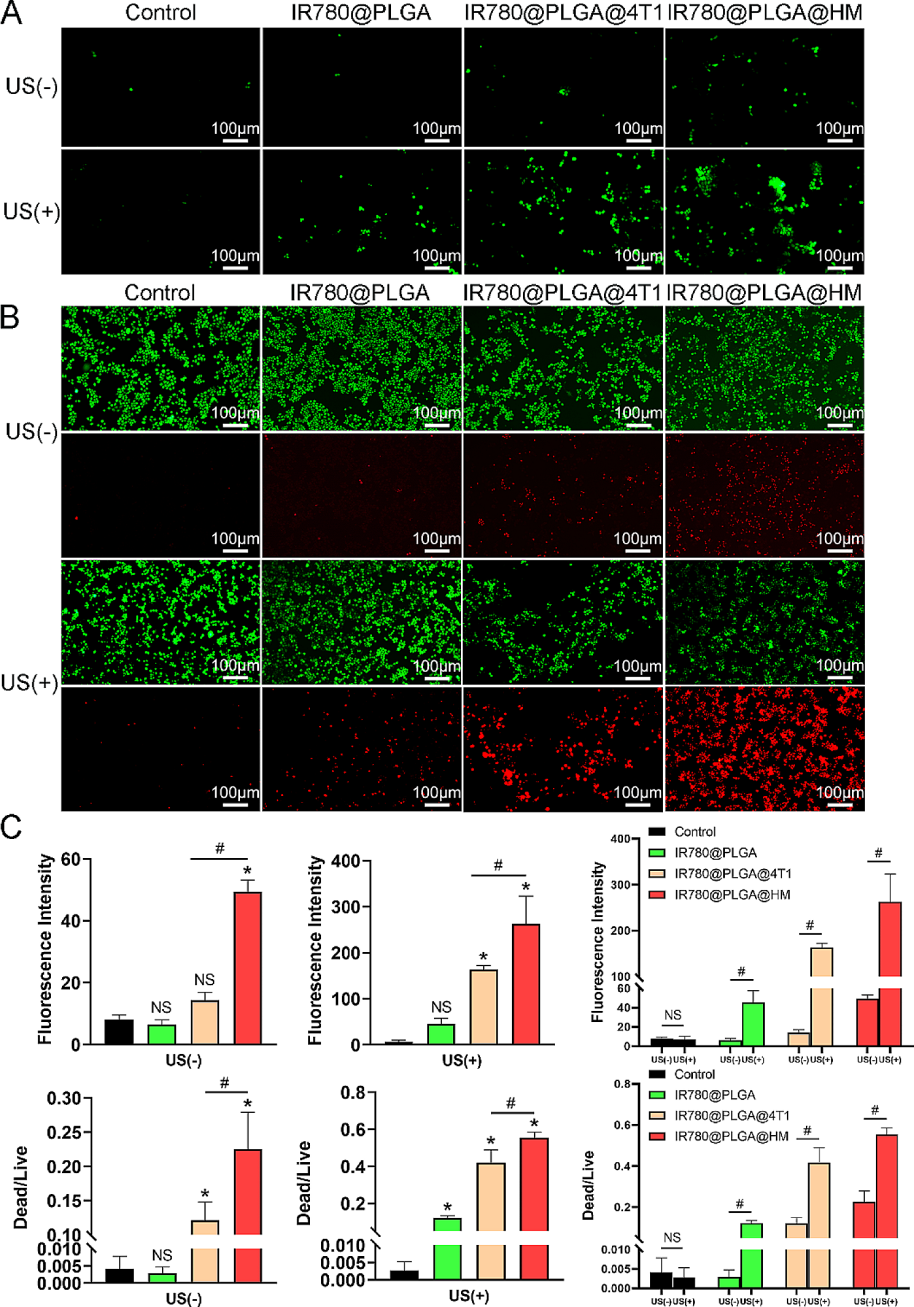
Evaluation of anti-tumor effects of nanoparticles in vitro. (**A**) ROS generation in 4T1 cells was detected by ROS probe after treatment of PBS, IR780@PLGA, IR780@PLGA@4T1, and IR780@PLGA@HM nanoparticles, with or without US treatment (1 W/cm^2^, 1 MHz, 10 s on and 10 s off for 2 minutes). DCFH-DA marked ROS as green fluorescence. (Scale bar = 100 μm). (**B**) 4T1 cell viability was visualized by live/dead staining after treatment. Live cells were stained as green and dead ones were red (Scale bar = 100 μm). (**C**) The fluorescence intensity of (**A**) and (**B**) was quantified. Results were presented as means SD (*n* = 3). Statistical significances were calculated via Student’s t-test, *p < 0.05 (compared with the control group). ^#^*p* < 0.05 (difference between compared groups). NS meant no significant difference

Since IR780 has been reported to be an effective ultrasound-responsive agent, we treated 4T1 cells with IR780-loaded nanoparticles and detected ROS generation to evaluate the sonodynamic effects of nanoparticles. When treated without ultrasound, 4T1 cells didn’t generate so much ROS in control, IR780@PLGA and IR780@PLGA@4T1 groups, with little green fluorescence observed (Fig. 5A). IR780@PLGA@HM increased ROS generation, which was significantly more than IR780@PLGA@4T1 nanoparticles and the control group (Fig. 5C), indicating that hybrid membrane could improve the ROS induction effect of IR780 loaded nanoparticles even without ultrasound irradiation. When treated with ultrasound irradiation, ROS generation was promoted in all IR780-loaded nanoparticle groups, while no promotion was seen in the ultrasound-treated control group (Fig. 5A). The fluorescence intensity of ROS was significantly stronger in IR780@PLGA@4T1 and IR780@PLGA@HM groups than control group. IR780@PLGA@HM induced more ROS generation than IR780@PLGA@4T1 nanoparticles (Fig. 5C). After comparing groups with and without ultrasound irradiation, it showed that ultrasound sharply increased ROS production in all IR780-loaded nanoparticles. After ultrasound treatment, the fluorescence intensity was 6.5 times more in IR780@PLGA group, 10.6 times more in IR780@PLGA@4T1 group, and 4.3 times more in IR780@PLGA @HM group. IR780@PLGA@HM nanoparticles with ultrasound promoted the most ROS generation of 4T1 cells (Fig. 5C). For one reason, more IR780@PLGA@HM nanoparticles were taken than other nanoparticles by 4T1 cells due to the better-targeting ability of HM coating, which improved the Sono-therapeutic effects of IR780@PLGA@HM nanoparticles. For another, OMV components would promote ROS generation of 4T1 cells. Therefore, IR780@PLGA@HM served as a good sonodynamic nanoparticle for tumor treatment. Then we evaluated the anti-tumor effects of nanoparticles on 4T1 cells in vitro. After nanoparticle treatment, we utilized live/dead staining to visualize 4T1 cell viability. We found that more 4T1 cells were stained as red in IR780@PLGA@4T1 and IR780@PLGA@HM groups compared with the control group when ultrasound was not exerted (Fig. 5B). The ratio of Dead/Live cells was significantly higher in these two groups, with IR780@PLGA@HM nanoparticles causing the most tumor cell death (Fig. 5C). After ultrasound irradiation, nanoparticles all led to decreased life and increased dead cells, with significantly raised Dead/Live ratios compared with the control group. IR780@PLGA@HM irradiated with ultrasound caused significantly more cell death than IR780@PLGA@4T1 nanoparticles (Fig. 5C). Comparison results showed that ultrasound treatment significantly increased the anti-tumor efficacy of nanoparticles, with the Dead/Live cells ratio increasing from 0.003 to 0.123, 0.121 to 0.417, and 0.225 to 0.554 in IR780@PLGA, IR780@PLGA@4T1, and IR780@PLGA@HM groups, respectively, while ultrasound didn’t increase cell death in the control group (Fig. 5C). It indicated that IR780@PLGA@HM nanoparticles with ultrasound irradiation could kill tumor cells efficiently. IR780@PLGA@HM nanoparticles were proven to be effective sonodynamic anti-tumor therapy in vitro.

**Fig. 6:**
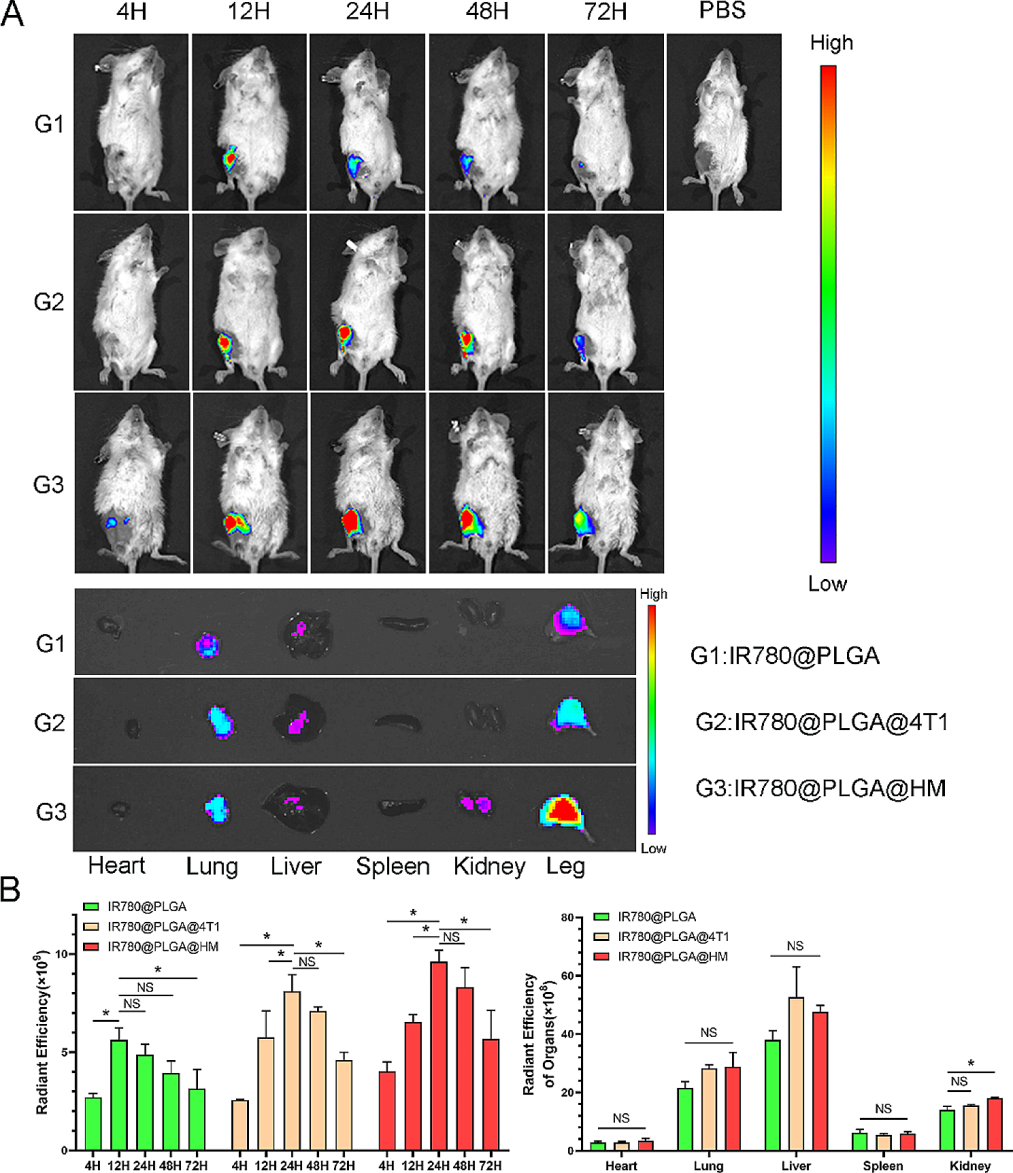
Evaluation of tumor-targeting capability of nanoparticles in vivo. (**A**) In vivo fluorescence images of tumor-bearing BALB/C mice were taken at 4 h, 12 h, 24 h, 48 h, and 72 h after intravenous injections of IR780@PLGA, IR780@PLGA@4T1 and IR780@PLGA@HM nanoparticles, respectively (*n* = 3). Then ex vivo fluorescence images of tumors and organs were photographed. (**B**) The fluorescence intensity of tumors at different time points and organs at 72 h were quantified. Statistical significances were calculated via Student’s t-test and One-way ANOVA test, *p < 0.05. NS meant no significant difference

To evaluate the targeting ability and distribution of nanoparticles in vivo, we injected IR780@PLGA, IR780@PLGA@4T1, and IR780@PLGA@HM nanoparticles into BALB/C mice and used the IVIS system to observe the distribution of nanoparticles at different time points, using PBS injection as negative control. Figure 6A and B revealed that 4T1 membrane and HM-coated nanoparticles gathered more in the tumor sites than naked IR780@PLGA nanoparticles at all time points. The fluorescence intensity of naked IR780@PLGA increased to a peak at 12 h and then presented a downtrend, with a radiant efficiency of less than 5 × 10^9^ after 24 h. IR780@PLGA@4T1 and IR780@PLGA@HM nanoparticles both reached the peak at 24 h and the fluorescence intensity was still larger than 5 × 10^9^ at 48 h in the IR780@PLGA@4T1 group and 72 h in IR780@PLGA@HM, indicating the improved targeting ability of 4T1 membrane and HM coating. Figure S6 showed that 24 h after injection, IR780@PLGA@HM nanoparticles presented the strongest fluorescence within tumor-bearing legs, which was no significantly higher than IR780@PLGA@4T1 and around twice the efficiency of naked IR780@PLGA nanoparticles. IR780@PLGA@HM showed the best tumor-targeting ability. For organ distribution, Fig. 6A and B showed that nanoparticles gathered more in the lungs and livers and less in the heart, spleen, and kidney, which was probably due to the reticuloendothelial system uptake. IR780@PLGA@HM nanoparticles gathered more in the kidney than the control group, with the difference in radiation efficiency as 4 × 10^8^, which could be explained by the prolonged circulation time caused by increased amounts of nanoparticles targeting tumor sites. There was no difference in radiant efficiency among groups in other organs.

**Fig. 7:**
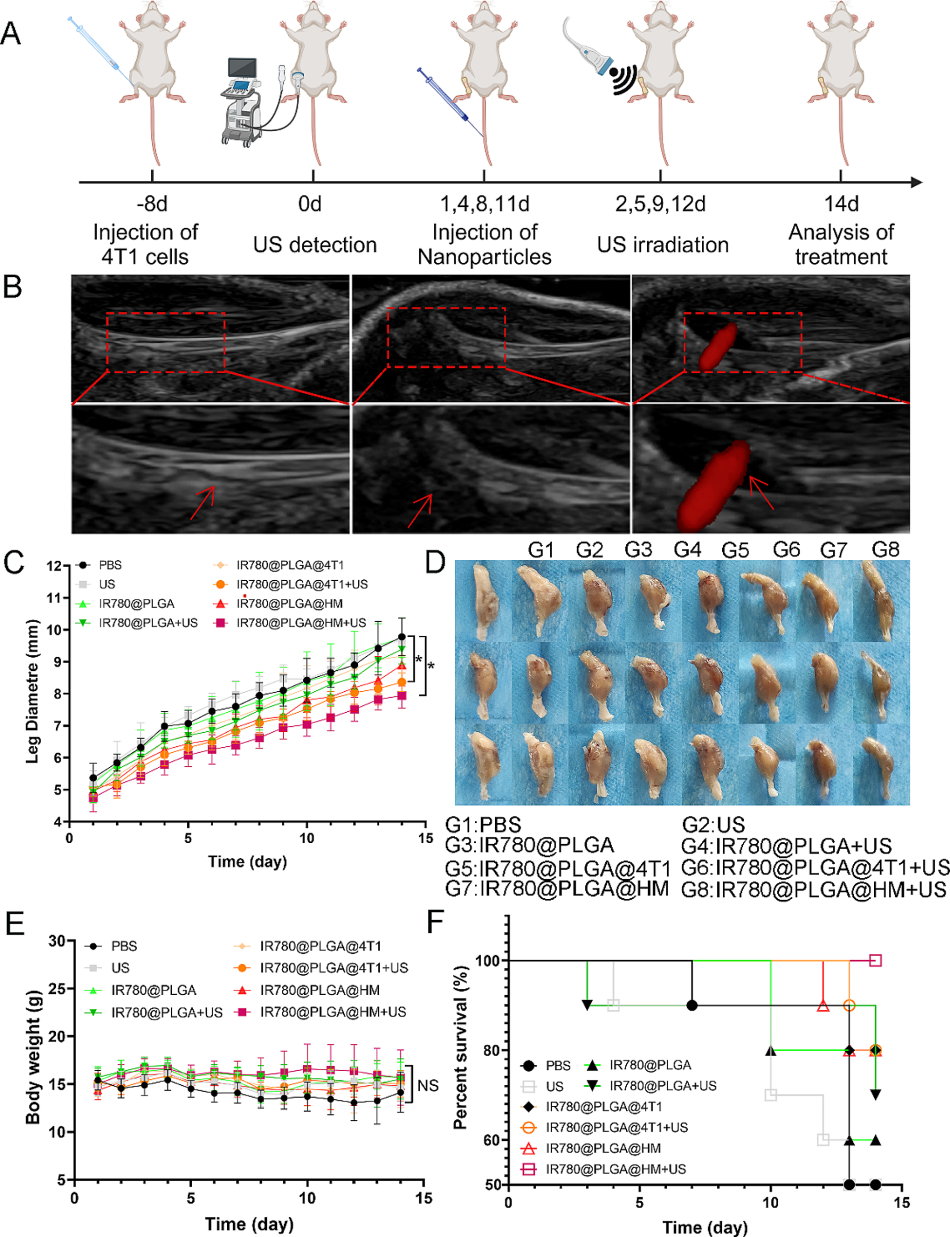
Evaluation of anti-tumor effects of nanoparticles in vivo. (**A**) Schematic illustration of in vivo test. (**B**) Ultrasound examination of established Breast Cancer Bone Metastasis animal models. The red rectangle showed the tibial plateau area. Red arrows marked bone and blood flow. (**C**) Leg diameters, (**D**) leg photos, (**E**) body weights, and (**F**) survival of mice were recorded during the process of treatment (*n* = 10). Statistical significances were calculated via Student’s t-test and One-way ANOVA test, *p < 0.05. NS meant no significant difference

After evaluating the in vitro anti-tumor, immune regulating, and in vivo tumor targeting ability, we further established Breast Cancer Bone Metastasis BALB/C mice models to evaluate the synergistic anti-tumor effects of nanoparticles. The in vivo test was conducted as Fig. 7A shows. Us irradiation was applied 24 h after nanoparticles injection, which was determined according to the peak time of nanoparticles detected in Fig. 6. Figure 7B showed that one week after the injection of tumor cells, an ultrasound examination revealed bone cortical discontinuity and bone integrity destruction in the tibial plateau, as well as locally enhanced blood flow signals marked by red arrows, indicating the successful establishment of Breast Cancer Bone Metastasis. After 2 weeks of treatment, Fig. 7C showed that the diameters of tumor-bearing legs increased in all treated groups. Leg diameters in PBS treated group increased from 5.37 mm to 9.75 mm, which was the biggest in all groups. The diameters in IR780@PLGA@4T1 with ultrasound and IR780@PLGA@HM with ultrasound groups were significantly smaller than PBS group, with 8.36 mm and 7.94 mm on the 14th day, respectively (Fig. 7C). The smaller legs of these two groups could also be seen in the morphological observation in Fig. 7D. Treatments in other groups didn’t significantly inhibit the growth of tumor-bearing legs when compared with the PBS group. As for body weight changes, no significant difference was observed among the different groups (Fig. 7E). 2 weeks after treatment, only 50% of mice were still alive in the control group and ultrasound-treated group. The percent survival was 100%, the highest in IR780@PLGA@HM with ultrasound group, and 80%, 80%, 80%, 70% and 60% in IR780@PLGA@HM, IR780@PLGA@4T1 with ultrasound, IR780@PLGA@4T1, IR780@PLGA with ultrasound and IR780@PLGA treated group, respectively. Figure S7 showed that IR780@PLGA@4T1 with ultrasound, IR780@PLGA@HM, and IR780@PLGA@HM with ultrasound treatment had a positive effect on inhibiting tumor further metastasis to lung, which could influence the survival of mice. These results demonstrated that IR780@PLGA@HM with ultrasound treatment effectively inhibited tumor growth and improved survival of Breast Cancer Bone Metastasis BALB/C mice.

**Fig. 8:**
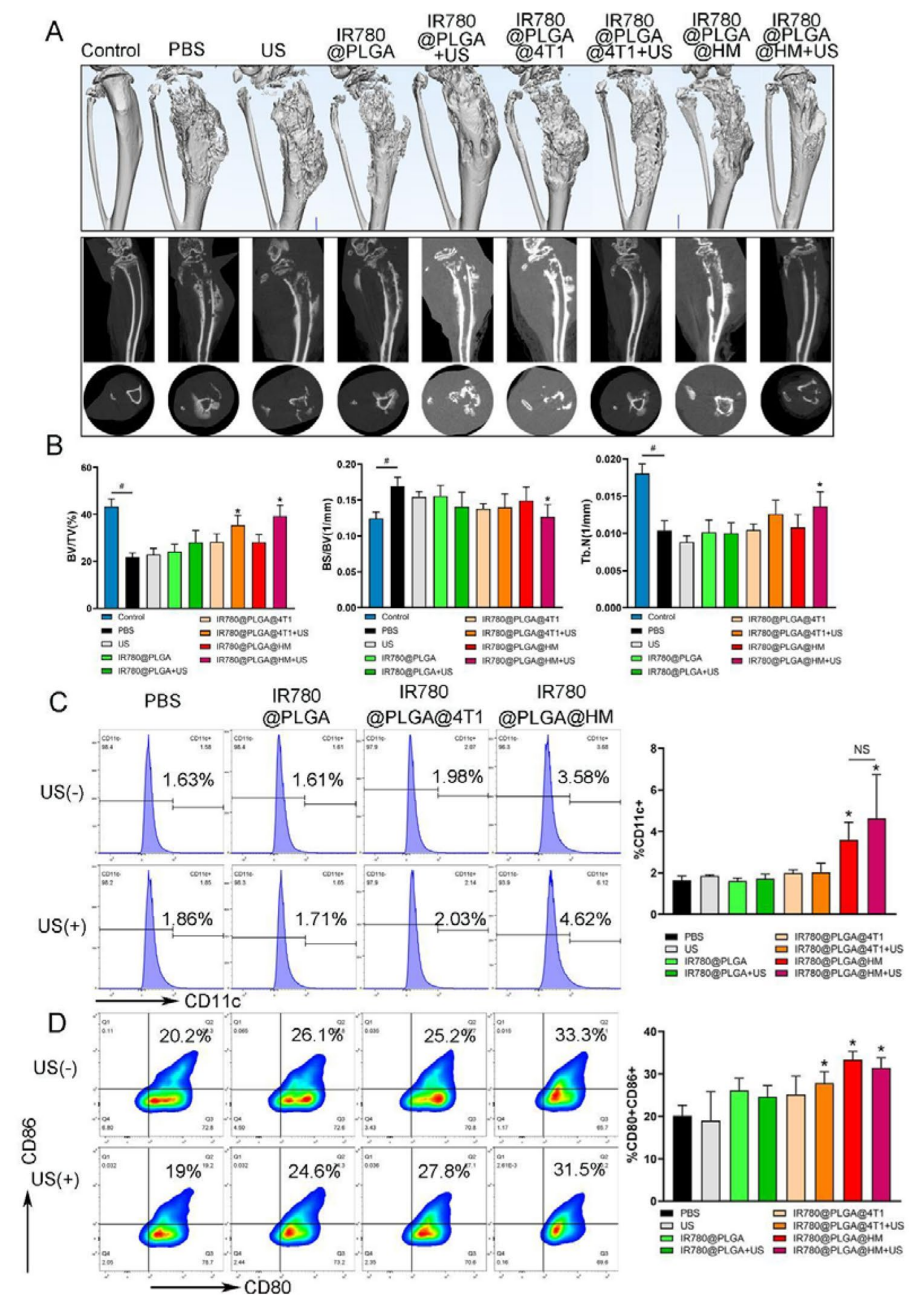
Nanoparticles inhibited bone destruction caused by Breast Cancer Bone Metastasis and promoted DC cell activation. (**A**) Micro CT images of tumor-bearing legs, with sagittal and transection views. (**B**) Quantification results of Bone Volume/ Tissue Volume (BV/TV), Bone Surface/Bone Volume (BS/BV), and Trabecular number (Tb.N) of the tibial plateau area in all groups. (**C**) Flow cytometry analysis results of CD11c positive cells of mice spleen after treatment, and (**D**) CD80 and CD86 positive cells were analyzed in the gate of CD11c positive cells. Statistical significances were calculated via Student’s t-test, *p < 0.05 (compared with the PBS group). ^#^*p* < 0.05 (difference between compared groups). NS meant no significant difference

As previously reported, breast cancer bone metastasis would destroy bone integrity and cause bone absorption, which could be observed and analyzed on Micro CT images [36]. Therefore, we acquired Micro CT images of tumor-bearing legs to evaluate the therapeutic effects of nanoparticles. Figure 8A presented 3D reconstruction images of legs, and sagittal and transection CT images of the tibial plateau, which showed that the right tibial bone was severely destroyed in PBS treated group, obviously different from the smooth surface and intact bone of the normal leg in the control group. CT images could see broken bone pieces and solar elastosis syndrome caused by tumors. After treatment, IR780@PLGA@HM with ultrasound performed the best in protecting bone from destruction, with the bone morphology less damaged than the other groups. Then we quantified the bone amount with several parameters. BV/TV, Tb. N, and Tb. Th reflected the amount of bone, while BS/BV and Tb. Sp would increase when bone tissue was destroyed and absorbed. As Fig. 8B showed, the tumor-bearing bone in the PBS group had decreased BV/TV score and Tb. N, and increased BS/BV ratio when compared with normal legs in the control group. However, IR780@PLGA@HM with ultrasound treatment significantly increased BV/TV and Tb. N, and decreased BS/BV when compared with the PBS group, demonstrating that to some extent, IR780@PLGA@HM with ultrasound significantly improved bone amount and inhibited bone loss caused by the tumor. IR780@PLGA@4T1 with ultrasound treatment also showed positive effects on improving BV/TV score, smaller than IR780@PLGA@HM. Figure S8 showed that the tumor increased Tb. Sp and didn’t affect Tb. Th in PBS group. No significant difference was found among all treatment groups on these two parameters, however, IR780@PLGA@HM with ultrasound treatment resulted in the smallest Tb. Sp and biggest Tb. Th. Therefore, IR780@PLGA@HM with ultrasound treatment had the best therapeutic effect on improving the bone amount and protecting the legs from tumor destruction.

Next, we detected activation of DC cells from mice spleen after nanoparticle treatment to evaluate the immune regulating effects. Activated DC cells were stained as CD11c + CD80 + CD86+. We selected the CD11c + cells from the whole cell population. After that, analyzed the proportion of CD80 + CD86 + cells in the gate of CD11c + cells. Figure 8C showed that IR780@PLGA@HM and IR780@PLGA@HM with ultrasound treatment significantly increased CD11c positive cells compared with the PBS group, from 1.63 to 3.58% and 4.62%, respectively, while no significant difference was found between these two groups. Under the CD11c positive gate, Fig. 8D showed that IR780@PLGA@4T1 with ultrasound treatment, IR780@PLGA@HM nanoparticles, and IR780@PLGA@HM with ultrasound treatment all significantly promoted CD80 and CD86 positive cells activation, from 20.2 to 27.8%, 33.3% and 31.5%, respectively. Therefore, IR780@PLGA@HM nanoparticles could promote DC cell activations with or without ultrasound treatment in vivo, while IR780@PLGA@4T1 nanoparticles with ultrasound treatment also had DC cell-activating function. IR780@PLGA@HM with ultrasound treatment had both anti-tumor and immune-regulating effects on Breast Cancer Bone Metastasis.

**Fig. 9:**
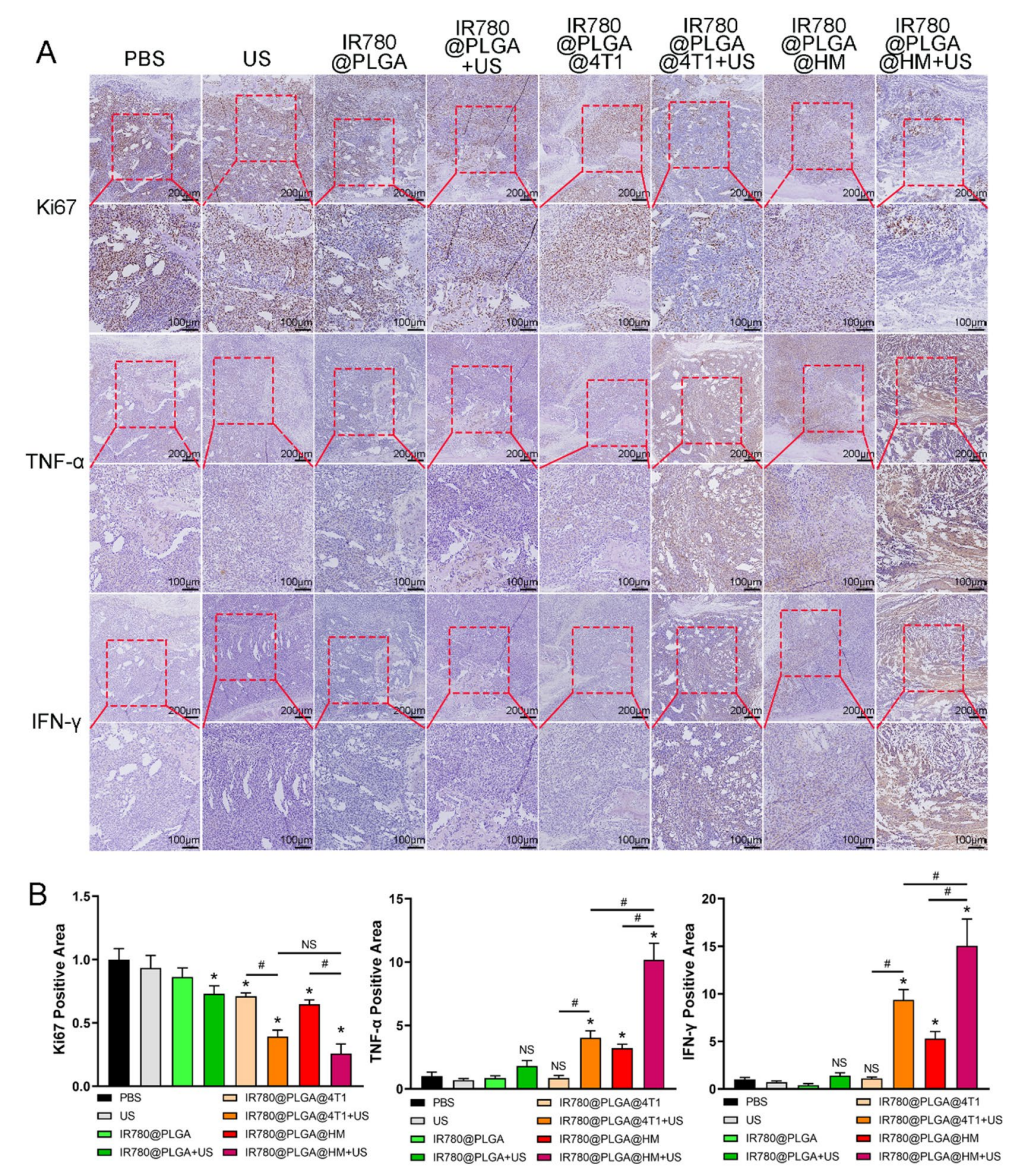
Nanoparticles inhibited 4T1 tumor cell proliferation and promoted inflammatory factor expression in tumor tissues. (**A**) Ki67, TNF-α, and IFN-γ expression in tumor tissues were detected by immunohistochemistry. The red rectangle shows the interested area. Scale bar = 200 μm, Scale bar = 100 μm. (**B**) Quantification results of Ki67, TNF-α, and IFN-γ expression. Statistical significances were calculated via Student’s t-test, *p < 0.05 (compared with the PBS group). ^#^*p* < 0.05 (difference between compared groups). NS meant no significant difference

We further detected nanoparticles’ therapeutic effects on tumors by HE staining and immunohistochemistry analysis. Figure S9 presented the morphological changes of the legs after 2-week treatment. It could be seen that the tibial bone was severely damaged by tumor erosion in the PBS group. IR780@PLGA@HM with ultrasound treatment led to smaller leg diameter and more intact bone structure than other groups, which was consistent with the Micro CT observations. Ki67 was detected to reflect 4T1 cell proliferation. TNF-α and IFN-γ were detected to evaluate immune regulation effects. Figure 9A and B showed that cell proliferation was significantly inhibited by IR780@PLGA with ultrasound, IR780@PLGA@4T1, IR780@PLGA@4T1 with ultrasound, IR780@PLGA@HM and IR780@PLGA@HM with ultrasound treatment. Ultrasound significantly strengthened the tumor-inhibiting effects of IR780@PLGA@4T1 and IR780@PLGA@HM nanoparticles. Although no significant difference was found between these two groups, IR780@PLGA@HM with ultrasound inhibited the most Ki67 expression in all groups. Then we analyzed the expression of inflammatory factors, TNF-α and IFN-γ, within tumor sites, and found that IR780@PLGA@4T1 with ultrasound, IR780@PLGA@HM and IR780@PLGA@HM with ultrasound treatment significantly promoted TNF-α and IFN-γ expression. Ultrasound irradiation also strengthened the immune regulating effects of IR780@PLGA@4T1 and IR780@PLGA@HM nanoparticles. IR780@PLGA@HM with ultrasound induced more TNF-α and IFN-γ expression than IR780@PLGA@4T1 with ultrasound group. Therefore, IR780@PLGA@HM with ultrasound treatment had the best therapeutic effects on inhibiting tumor cell proliferation and promoting inflammatory factor expression.

**Fig. 10:**
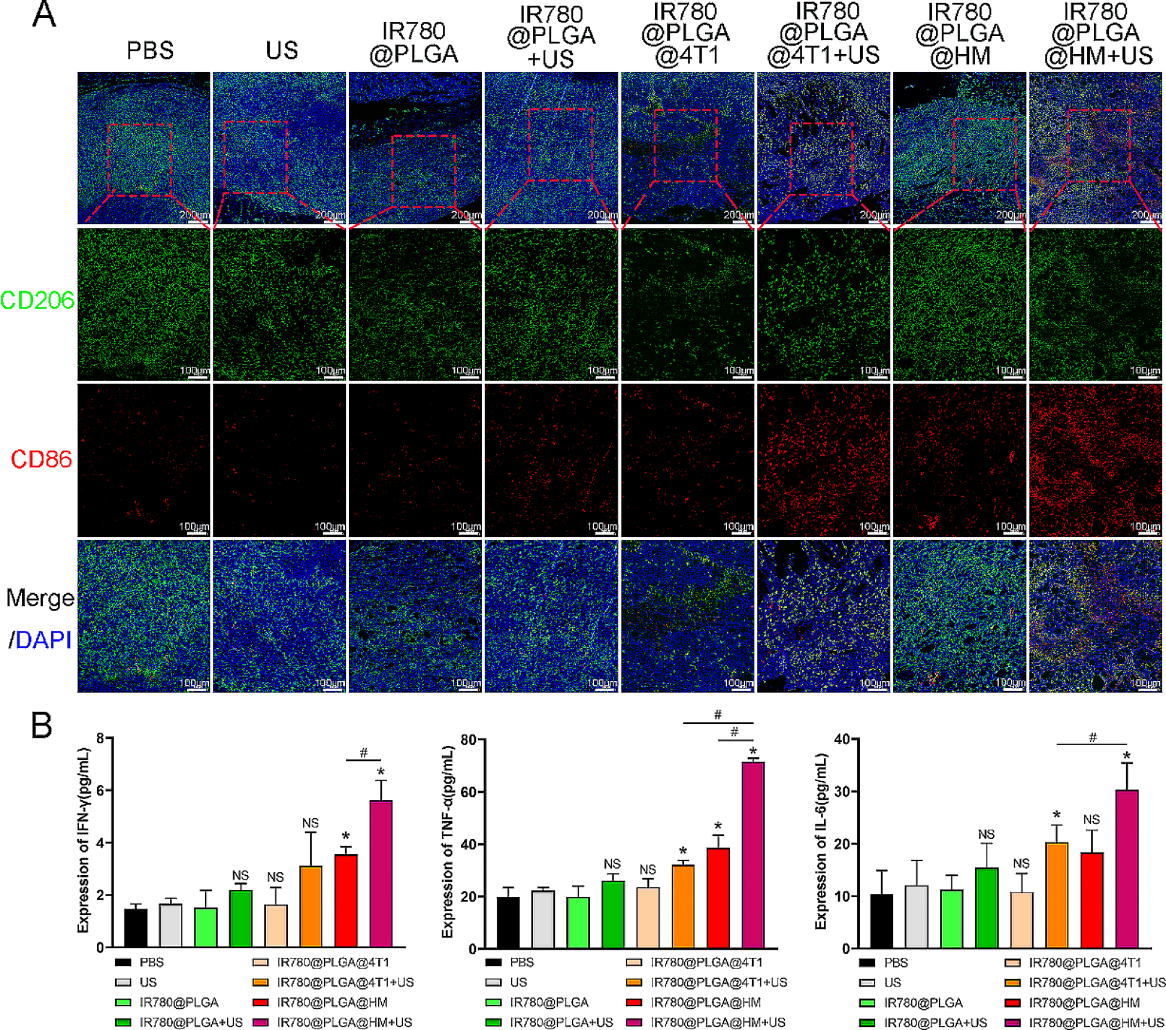
Evaluation of macrophage polarization in vivo and inflammatory cytokines expression in serum. (**A**) CD86 and CD206 expression in tumor tissues were detected by immunofluorescence staining. The nucleus was stained blue by DAPI. CD86 and CD206 were marked as red and green, respectively. The red rectangle shows the interested area. Scale bar = 200 μm, Scale bar = 100 μm. (**B**) IFN-γ, TNF-α, and IL-6 in serum were detected. Statistical significances were calculated via Student’s t-test, *p < 0.05 (compared with the PBS group). #*p* < 0.05 (difference between compared groups). NS meant no significant difference

Finally, we detected macrophage polarization within tumors and evaluated inflammatory cytokines expression in serum to further improve the immune regulating effect of nanoparticles. Figure 10A and Figure S10 showed that CD206 expressed more than CD86 in tumor tissues under PBS treatment, with a CD86/CD206 ratio smaller than 0.1, while more CD86 expression could be seen in IR780@PLGA@4T1 with ultrasound and IR780@PLGA@HM with ultrasound groups. Quantification results showed that IR780@PLGA with ultrasound, IR780@PLGA@4T1 with ultrasound, IR780@PLGA@HM, and IR780@PLGA@HM with ultrasound treatment significantly raised the ratio compared with the PBS group. Ultrasound irradiation significantly improved IR780@PLGA@4T1 and IR780@PLGA@HM nanoparticles’ ability to raise the CD86/CD206 ratio. IR780@PLGA@HM with ultrasound treatment performed the best in improving CD86/CD206 ratio, larger than 1, in all groups (Figure S10). Figure 10B showed that IR780@PLGA@HM with ultrasound treatment significantly raised IFN-γ, TNF-α, and IL-6 expression in serum. IR780@PLGA@HM also increased IFN-γ and TNF-α expression and IR780@PLGA@4T1 with ultrasound treatment increased TNF-α and IL-6 levels in serum. Therefore, we could conclude that IR780@PLGA@HM with ultrasound treatment effectively promoted macrophage type I polarization within tumor tissues and inflammatory cytokines expression in Breast Cancer Bone Metastasis BALB/C mice.

## Conclusions

In conclusion, the constructed IR780@PLGA@HM nanoparticles showed satisfied therapeutic effects on breast cancer bone metastasis in both vitro and vivo experiments. HM, infused with the cancer cell membrane and OMV, enabled nanoparticles to target tumor cells and regulate immune response simultaneously. The loaded IR780 provided excellent ultrasound sensitivity to generate ROS. Therefore, in vitro, the IR780@PLGA@HM nanoparticles efficiently targeted 4T1 cells, promoted macrophage type I polarization, increased anti-tumor inflammatory factors expression, and induced sufficient ROS to kill tumor cells with ultrasound irradiation. In vivo, the IR780@PLGA@HM nanoparticles with ultrasound treatment effectively improved the survival of mice, protected bone from 4T1 tumor destruction, promoted spleen DC cell activation, and increased macrophage type I polarization and anti-tumor inflammatory factors expression. The nanoparticles combined sonodynamic therapy with immune regulation therapy well, with good biosafety. The proposed strategy could also be adapted to other diseases, providing a novel approach to combining immunotherapy with other therapies to achieve a synergetic and satisfactory therapeutic effect.

## Methods and materials

### Bacterial strains, cells, and animals

*E. coli DH5α* is preserved in the bacteria library of our laboratory and cultured in Luria-Bertani (LB) Broth (BKMAN, Changsha, China). Murine mammary carcinoma 4T1, B16 melanoma, and human osteosarcoma U2OS cells were acquired from Procell (Wuhan, China). RAW264.7 and L929 cells were sourced from Xiangya Hospital of Central South University (Changsha, China). Female BALB/C mice (4–6 weeks old, 15–18 g) were supplied by Hunan SJA Laboratory Animal Company and housed in the Department of Laboratory Animals at Central South University. Primary bone marrow-derived macrophages (BMDM) were harvested from female BALB/C mice (4–6 weeks) and cultured in DMEM/F12 (Procell, Wuhan, China) containing 10% FBS and 25 ng/mL murine macrophage colony-stimulating factor (M-CSF) (R&D Systems, USA). All animal experiments were conducted following the ARRIVE guidelines and were approved by the Ethics Committee of Xiangya Hospital at Central South University. (202,110,140).

### 4T1 membrane and OMVs preparation

The 4T1 cell membrane was obtained as previously described [37, 38]. Briefly, 4T1 cells were harvested when the cell density reached 80–90% on cell dishes. The cells were washed twice with PBS by centrifugation at 1,500 g for 5 min. Subsequently, cells were resuspended in double-distilled water and the cell membrane was disrupted using an ultrasound processor (Sonic and Materials Inc., USA) operating at 20 kHz and 130 W, with 5 s on and 5 s off for 5 min, while kept on ice.

Following the disruption, the cell membrane was extruded through a 200 nm polycarbonate membrane in a liposome extruder (Avestin, Germany) for 20 cycles to standardize the membrane particle size. The suspension was then centrifuged at 15,000 g for 30 min to obtain 4T1 cell membrane vesicles. The protein content of the cell membrane was measured using a BCA kit, and the vesicles were stored at -80℃.

*E. coli* strains are commonly used for producing OMVs in tumor immune treatment [28, 34, 39]. In this study, *E. coli DH5α* was cultured for OMV production [30, 32]. Following established protocols [37], *E. coli DH5α* was cultured in LB Broth in a shaking incubator at 37℃. The bacterial suspension was collected when the medium’s OD_600nm_ reached 1.5 and then centrifuged at 5,000 rpm for 5 min at 4℃. The supernatant was extruded through a 200 nm filter twice to remove residual bacteria.

To obtain OMVs, the supernatant was added to an Amicon Ultra15 centrifugal filter tube (10 kDa; Millipore, USA) and centrifuged at 1,500 g for 20 min. The concentrated liquid was transferred to a new tube, and 200 µL of Exoquick TC (System Biosciences, Bay Area, California, USA) was added. The mixture was incubated at 4℃ for 12 h and then centrifuged at 1,500 g at 4℃ for 30 min. The precipitate obtained was OMVs. OMVs were resuspended in PBS, and the protein amount was measured using a BCA kit. The OMVs were then stored at -80℃.

### Construction of the 4T1-OMVs hybrid membrane

The hybrid membrane was constructed by infusing 4T1 cell membrane and OMVs at a 1:1 weight ratio. The membrane weight was considered twice as much as the protein weight measured above [32]. The membrane suspension was mixed and placed into an ultrasonic bath at 37 ℃ for 20 min, using an ultrasonic cleaner (Granbo Sonic, Shenzhen, China). The suspension was then extruded through a 200 nm polycarbonate membrane 11 times to physically infuse the membrane. Finally, the mixture was centrifuged at 12,000 rpm for 30 min to obtain the hybrid membrane of 4T1 cells and OMVs [28].

To identify the successful construction, 4T1 membrane, OMVs, and hybrid membrane were observed using a transmission electron microscope (TEM, Hitachi H-7600). Particle sizes and zeta potentials were determined using a dynamic light scattering (DLS) analyzer (Malvern Nano ZS, UK). Western blot analysis was employed to verify the protein components of HM, utilizing anti-VCAM-1 (Abclonal, A19131, Rabbit, 110 KD) and anti-OMPC (abbexa, abx243143, Rabbit, 60 KD). 4T1 membrane and OMVs were labeled with DiO and DiI (Beyotime, Shanghai, China), respectively, before being mixed. Following construction, the labeled HM was cultured with 4T1 cells for 4 h and visualized using confocal laser scanning microscopy (CLSM, Carl Zeiss, LSM 510 META).

### Construction of IR780@PLGA@HM nanoparticles

IR780-loaded PLGA nanoparticles (referred to as IR780@PLGA) were constructed using a single emulsion evaporation protocol, protected from light [38, 40, 41]. PLGA, IR780 iodide, and polyvinyl alcohol (PVA) were purchased from Sigma Aldrich (St. Louis, MO, USA). To initiate the process, 100 mg of PLGA and 1 mg of IR780 iodide were dissolved in 3 mL of dichloromethane. After complete dissolution, 10 mL of pre-cooled PVA solution (4% w/v), previously chilled at 4 °C, was added to the solution. An ultrasonic processor (Sonics, VCX150, USA) was employed to emulsify the mixture for 4 min with a 5-second on-and-off cycle. Subsequently, the emulsified solution was introduced into 30 mL of double-deionized water and stirred at room temperature for 3 h to allow for evaporation. The resulting solution was collected, and centrifuged at 10,000 rpm for 7 min, and after washing the centrifuged precipitate twice with double deionized water, the IR780@PLGA nanoparticle sediment was obtained.

The 4T1 cell membrane and HM were both utilized in the construction of IR780@PLGA@4T1 and IR780@PLGA@HM nanoparticles, respectively. A 500 µL solution of IR780@PLGA (1 mg/mL) was mixed with a 500 µL membrane solution (1 mg/mL) and sonicated for 10 min to coat the membrane onto IR780@PLGA. The solution was then centrifuged at 8,000 rpm for 5 min to remove any uncoated membrane. The deposit was washed twice with double-deionized water to obtain the final membrane-coated nanoparticles. The nanoparticles were redispersed in 1 mL PBS for further detection and characterization.

### Characterization of IR780@PLGA@HM nanoparticles

To identify the coating, IR780@PLGA, and IR780@PLGA@HM were observed with TEM and analyzed by DLS. Western blotting was also employed to verify the specific components of HM on the particles. Additionally, HM was labeled with DiO, IR780@PLGA nanoparticles were labeled with DiI, and IR780@PLGA@HM were visualized using CLSM. The presence of IR780 within IR780@PLGA and IR780@PLGA@HM nanoparticles was assessed using a UV-Vis-NIR spectrophotometer (Cary 5000, USA).

IR780 loading and encapsulation efficiencies were calculated according to the following equation:

$$\text{Drug\;loading }\left(\text{\%}\right)=\frac{\begin{array}{l}{\text{Weight\;of\;IR780\;loaded}}\\{\text{\;in\;the\;nanoparticles}}\end{array}}{\text{Weight\;of\;nanoparticles}}\times 100\%$$

$$\text{Encapsulation\;efficiency }\left(\text{\%}\right)=\frac{\begin{array}{l}{\text{Weight\;of\;IR780\;loaded}}\\{\text{\;in\;the\;nanoparticles}}\end{array}}{\text{Weight\;of\;added\;IR780}}\times 100\%$$

To assess the stability of IR780@PLGA@HM nanoparticles, placed them in PBS buffer at 4℃ for one week, with daily measurements of their particle size and Polydispersity Index (PDI). A fluorescent probe, Singlet Oxygen Sensor Green (SOSG), was employed to quantify reactive oxygen species (ROS) production. In a quartz cuvette, 100 µL of IR780@PLGA@HM nanoparticle solution at varying concentrations (0.5, 1, 1.5, 2 mg/mL) and 1 µL of SOSG (1 mM) were mixed. The ROS generation was induced by irradiating the mixture using a low-frequency US transducer (WED-100, WELLD Medical Electronics, China) at 2 W/cm^2^, 1 MHz, and 50% duty cycle for 30 s. Subsequently, the fluorescence spectra of SOSG were recorded on a fluorescence spectrometer, with an excitation wavelength of 504 nm. To investigate the time-dependent ROS production, 1.5 mg/mL of IR780@PLGA@HM nanoparticles were subjected to ultrasound treatment for 40, 60, and 120 s.

### Evaluation of biosafety of IR780@PLGA@HM nanoparticles in vitro and in vivo

RAW264.7 and L929 cells were employed to evaluate the toxicity of IR780@PLGA@HM in vitro. Briefly, cells were seeded in 96-well plates at a density of 5,000 cells per well. After 24 h, the cells were treated with different concentrations of IR780@PLGA@HM (0, 500, 1,000, 1,500, 2,000 µg/mL). Following 24 and 48 h of incubation, cell viability was assessed using the Cell Counting Kit-8 (CCK8) Kit (New Cell & Molecular Biotech Co., Ltd., Suzhou, China). To evaluate the sonodynamic cytotoxicity of nanoparticles, 24 h after IR780@PLGA@HM treatment, cell culture was removed and cells were washed with PBS 3 times to remove the untaken nanoparticles. Then cells were treated with ultrasound (1 W/cm2, 1 MHz, 10 s on and 10 s off for 2 min). After that, cell viability was evaluated using the CCK8 Kit.

Furthermore, a hemolysis experiment was conducted to evaluate the biosafety of nanoparticles. Fresh RBCs were collected from a BALB/C mouse by centrifuging blood and washing the cells with PBS three times. The RBCs were then resuspended in PBS, and 0.25 mL of a 0.2% RBC solution (v/v) was added to 0.75 mL of nanoparticle solution with different concentrations (final concentrations: 0, 500, 1,000, 1,500, 2,000 µg/mL). After incubating for 0.5, 1, 2, and 3 h, the solution was centrifuged, and the absorbance of supernatants at 540 nm was measured. RBCs treated with deionized water served as a positive control.

To evaluate biosafety in vivo, BALB/C mice were intravenously injected with 200 µL of PBS or 10 mg/mL of IR780@PLGA@HM twice a week (*n* = 3 per group). 2 weeks later, the mice were euthanized to collect blood for analysis. Detected a complete blood count to reflect the quantity of various types of blood cells. Aspartate aminotransferase (AST), Alanine aminotransferase (ALT), Alkaline phosphatase (ALP), and Total protein (TP) were assessed to reflect hepatic function, while Blood Urea Nitrogen (BUN) and Uric acid (UA) were measured as an indicator of kidney function. Heart, lung, liver, spleen, and kidney were collected for HE staining to observe the toxicity of nanoparticles on organs.

### Evaluation of targeting ability of IR780@PLGA@HM nanoparticles in vitro

CLSM observation was employed to assess the targeting ability of nanoparticles, while flow cytometry was utilized to quantify cell uptake. The homotypic targeting ability of nanoparticles was evaluated in the following groups: IR780@PLGA, IR780@PLGA@4T1, and IR780@PLGA@HM (*n* = 3 per group). The concentration of nanoparticles was 1000 µg/mL.

For CLSM observation, 4T1, B-16, and U2OS cells were seeded in confocal dishes at a density of 50,000 cells per dish and cultured for 24 h. IR780@PLGA nanoparticles labeled with DiI and the cell membrane with DiO during construction were added to the culture medium of 4T1 cells at a concentration of 1000 ug/mL and incubated for 1, 2, and 4 h. Subsequently, free nanoparticles were washed out with PBS, cells were stained with DAPI, and observation was conducted with CLSM. B-16 and U2OS cells were incubated with IR780@PLGA@HM (1000 ug/mL) for 2 h and detected using CLSM.

To discuss the targeting ability of IR780@PLGA@HM towards RAW264.7 cells and 4T1 cells, treated cells with IR780@PLGA@HM (1000 ug/mL) for 4 h and detected them using CLSM. Nanoparticles were labeled with DiI and cells were stained with DAPI.

For flow cytometry detection, 4T1, B-16, and U2OS cells were seeded in 6-well plates at a density of 150,000 cells per well. After 24 h, 4T1 cells were treated with different nanoparticles for 1, 2, and 4 h. B-16 and U2OS cells were incubated with IR780@PLGA@HM (1000 ug/mL) for 2 h. Subsequently, cells were harvested and their fluorescence at 780 nm was measured using a flow cytometer.

### Evaluation of macrophage polarization in vitro

RAW264.7 cells were utilized to assess the impact of IR780@PLGA@HM on macrophage polarization. The cells were plated in 12-well plates, and when the density reached 60%, they were treated in various groups (n = 3 per group): control medium, OMV, IR780@PLGA, IR780@PLGA@4T1, and IR780@PLGA@HM. The concentration of nanoparticles was 1000 µg/mL. After 24 hours, the cells were washed with PBS twice and collected for analysis. Flow cytometry was employed to quantify the M1 polarization proportion. Primary BMDM cells were harvested and also treated with different nanoparticles as described above. After 24 hours, detected the M1 polarization proportion of BMDM using flow cytometry. RAW264.7 cells were stained with PE anti-mouse CD86 Antibody (Biolegend, Beijing, China) and APC anti-mouse F4/80 Antibody (Biolegend, Beijing, China) before being analyzed in a flow cytometer. RNA extraction was performed using Trizol (Yeasen, China). Following the measurement of RNA concentration, the expression levels of CD86, CD206, IL-6, TNF-α, and IFN-γ in different groups were assessed using the RT-qPCR SYBR Green Kit (Yeasen, China). The primer sequences used are listed below (5’ to 3’):

CD86 F: TCTCCACGGAAACAGCATCT,

CD86 R: CTTACGGAAGCACCCACGAT.

CD206 F: CCTATGAAAATTGGGCTTACGG,

CD206 R: CTGACAAATCCAGTTGTTGAGG.

IL-6 F: ATCCAGTTGCCTTCTTGGGACTGA,

IL-6 R: TTGGATGGTCTTGGTCCTTAGCCA.

TNF-Α F: AGCCGATGGGTTGTACCTTG,

TNF-α R: ATAGCAAATCGGCTGACGGT.

IFN-γ F: ATGAACGCTACACACTGCATC,

IFN-γ R: CCATCCTTTTGCCAGTTCCTC.

The culture medium from the cells was collected to assess the expression levels of various cytokines, including IL-6, TNF-α, and IFN-γ, using Cytometric Bead Array (CBA).

### In vitro anti-tumor efficacy

4T1 cells were seeded in a 12-well plate at a density of 50,000 cells per well. After 24 h, cells were treated with different nanoparticles and subjected to ultrasound. Based on the difference in nanoparticles and ultrasound (US) conditions, the tumor cells were divided into 8 groups: US (-) with control (PBS), IR780@PLGA, IR780@PLGA@4T1, and IR780@PLGA@HM; US (+) with control (PBS), IR780@PLGA, IR780@PLGA@4T1, and IR780@PLGA@HM (*n* = 3 per group). The concentration of nanoparticles was 1000 µg/mL. After 4 h of treatment, the US (+) group underwent low-frequency ultrasound (1 W/cm^2^, 1 MHz, 10 s on and 10 s off) for 2 minutes, while the US (-) group did not receive ultrasound treatment.

For cell viability assessment, the culture medium was removed, cells were washed twice with PBS and then stained with the Calcein-AM/PI Double S7 stain kit (Yeasen, Shanghai, China) at 37 °C for 15 min. Live cells were stained green, and dead cells were stained red. The Leica fluorescence microscope (Leica Microsystems) was used to observe the survival status of tumor cells. For ROS detection, the Reactive Oxygen Species Assay Kit (Yeasen, Shanghai, China) was used. After removing the medium, cells were washed twice with PBS, and then stained with a working solution of DCFH-DA at a concentration of 10 mM in serum-free medium at 37 °C for 30 min. Subsequently, the Leica fluorescence microscope was employed to observe ROS production within cells, which appeared green under excitation at 488 nm.

### Establishment of an animal model of breast Cancer bone metastasis

After a one-week acclimatization to the new environment, female BALB/C mice aged 4–6 weeks were used to establish the animal model. Following anesthesia, the skin surrounding the tibial plateau was removed. Using a 1 mL syringe needle, a hole was drilled into the tibial plateau, directed towards the distal end of the tibia. Subsequently, a 20 µL suspension containing 4T1 cells (with a cell count of 1 × 10^5^) was injected into the tibial plateau using an insulin needle. Postoperatively, the wound status was monitored daily. One week after the surgery, ultrasound was employed to assess the tibia, evaluating the continuity of the bone cortex and the surrounding blood flow to confirm the success of the modeling. Breast cancer bone metastasis occurred approximately one week after the surgery [36].

### In vivo distribution of IR780@PLGA@HM nanoparticles

To assess the targeting efficacy of IR780@PLGA@HM nanoparticles towards 4T1 tumors, BALB/C mice bearing 4T1 bone metastasis received intravenous injections of 200 µL of 10 mg/mL solutions containing different nanoparticles (*n* = 3 per group): IR780@PLGA, IR780@PLGA@4T1, and IR780@PLGA@HM. In vivo fluorescence was monitored at various time intervals (4 h, 12 h, 24 h, 48 h, and 72 h post-administration) using a Lumina IVIS Spectrum imaging system (PerkinElmer, USA). After 72 h, the limbs and major organs were harvested and subjected to ex vivo fluorescence imaging to assess nanoparticle distribution. The IVIS system was utilized to quantify fluorescence intensity.

### In vivo anti-tumor efficacy

Tumor-bearing mice were randomly assigned to eight treatment groups: PBS, US, IR780@PLGA, US + IR780@PLGA, IR780@PLGA@4T1, US + IR780@PLGA@4T1, IR780@PLGA@HM, US + IR780@PLGA@HM (*n* = 10 per group). Following the successful modeling, nanoparticles were administrated twice a week, intravenously at a dose of 200 µL with a concentration of 10 mg/mL. Ultrasound was applied one day after nanoparticles treatment, with parameters as 1 W/cm^2^, 1 MHz, with a 10-second on and 10-second off cycle for 2 minutes. Mice survival, leg circumference, and body weight were monitored and recorded every three days.

After a two-week treatment period, mouse legs, main organs, and blood samples were collected to evaluate treatment outcomes. Micro-computed tomography (micro-CT, Viva CT-80) scans of the legs were performed, and SkyScan CT analysis software (SCANCO Medical AG, Zurich, Switzerland) was utilized to analyze percent bone volume (bone volume/tissue volume, BV/TV), bone surface/bone volume ratio (BS/BV), Trabecular number (Tb. N), Trabecular separation (Tb. Sp) and Trabecular thickness (Tb. Th).

Morphological changes in the mouse tibia were assessed through Hematoxylin and eosin (HE) staining. Ki67, TNF-α, and IFN-γ expression were detected by immunohistochemistry to reflect tumor growth and inflammatory regulation. Macrophage polarization was detected by immunofluorescence staining, with CD86 and CD206. Blood samples underwent CBA to detect anti-tumor cytokines expression. Spleen tissues were collected for flow cytometry analysis to investigate dendritic cell (DC) maturation.

### Statistical analysis

All results were shown as the mean values ± standard deviations after repeating at least three times. One-way ANOVA and Student’s t-test were adapted to analyze the data by using GraphPad Prism 8.0.1 software. *p < 0.05 and #*p* < 0.05 were considered statistically significant.

### Electronic supplementary material

Below is the link to the electronic supplementary material.

Supplementary Material 1

## Data Availability

All data generated or analyzed during this study are included in this published article.
