# Correction: Bacterial outer membrane vesicle-cancer cell hybrid membrane-coated nanoparticles for sonodynamic therapy in the treatment of breast cancer bone metastasis

**DOI:** 10.1186/s12951-024-02649-4

**Published:** 2024-07-10

**Authors:** Jiahao Wang, Shuailong Liang, Sijie Chen, Tianliang Ma, Mingyu Chen, Chengcheng Niu, Yi Leng, Long Wang

**Affiliations:** 1 Department of Orthopedics, Xiangya Hospital, Central South University, Changsha, China; 2 The School of Medicine, Nankai University, Tianjin, 300071 China; 3 Hunan Engineering Research Center of Biomedical Metal and Ceramic Implants, Xiangya Hospital, Central South University, Changsha, China; 4 National Clinical Research Center for Geriatric Disorders, Xiangya Hospital, Central South University, Changsha, China; 5 Department of Ultrasound Diagnosis, Second Xiangya Hospital, Central South University, Changsha, China; 6 Department of Rehabilitation, Xiangya Hospital, Central South University, Changsha, China; 7 Hunan Key Laboratary of Aging Biology, Xiangya Hospital, Central South University, 87 Xiangya Road, Kaifu District, Changsha, 410008 Hunan China

**Correction: Journal of Nanobiotechnology (2024) 22:328** 10.1186/s12951-024-02619-w

In this article, the affiliation details of all the authors except the authors Mingyu Chen and Chengcheng Niu were incorrectly given as:

1. The School of Medicine, Nankai University, Tianjin 300071, China
2. Hunan Engineering Research Center of Biomedical Metal and Ceramic Implants, Xiangya Hospital, Central South University, Changsha, China
3. National Clinical Research Center for Geriatric Disorders, Xiangya Hospital, Central South University, Changsha, China
4. Department of Orthopedics, Xiangya Hospital, Central South University, Changsha, China

But should have been

1. Department of Orthopedics, Xiangya Hospital, Central South University, Changsha, China
2. The School of Medicine, Nankai University, Tianjin 300071, China
3. Hunan Engineering Research Center of Biomedical Metal and Ceramic Implants, Xiangya Hospital, Central South University, Changsha, China
4. National Clinical Research Center for Geriatric Disorders, Xiangya Hospital, Central South University, Changsha, China

The original article has been corrected.

