# Supplementary material for: Bacterial outer membrane vesicle-cancer cell hybrid membrane-coated nanoparticles for sonodynamic therapy in the treatment of breast cancer bone metastasis
Source: J Nanobiotechnology. 2024 Jun 10;22:328. doi: 10.1186/s12951-024-02619-w (PMC11165797; doi:10.1186/s12951-024-02619-w)
Supplement: Supplementary file 1 — Supplementary Material 1 [file 12951_2024_2619_MOESM1_ESM.docx]

**Supplementary materials**


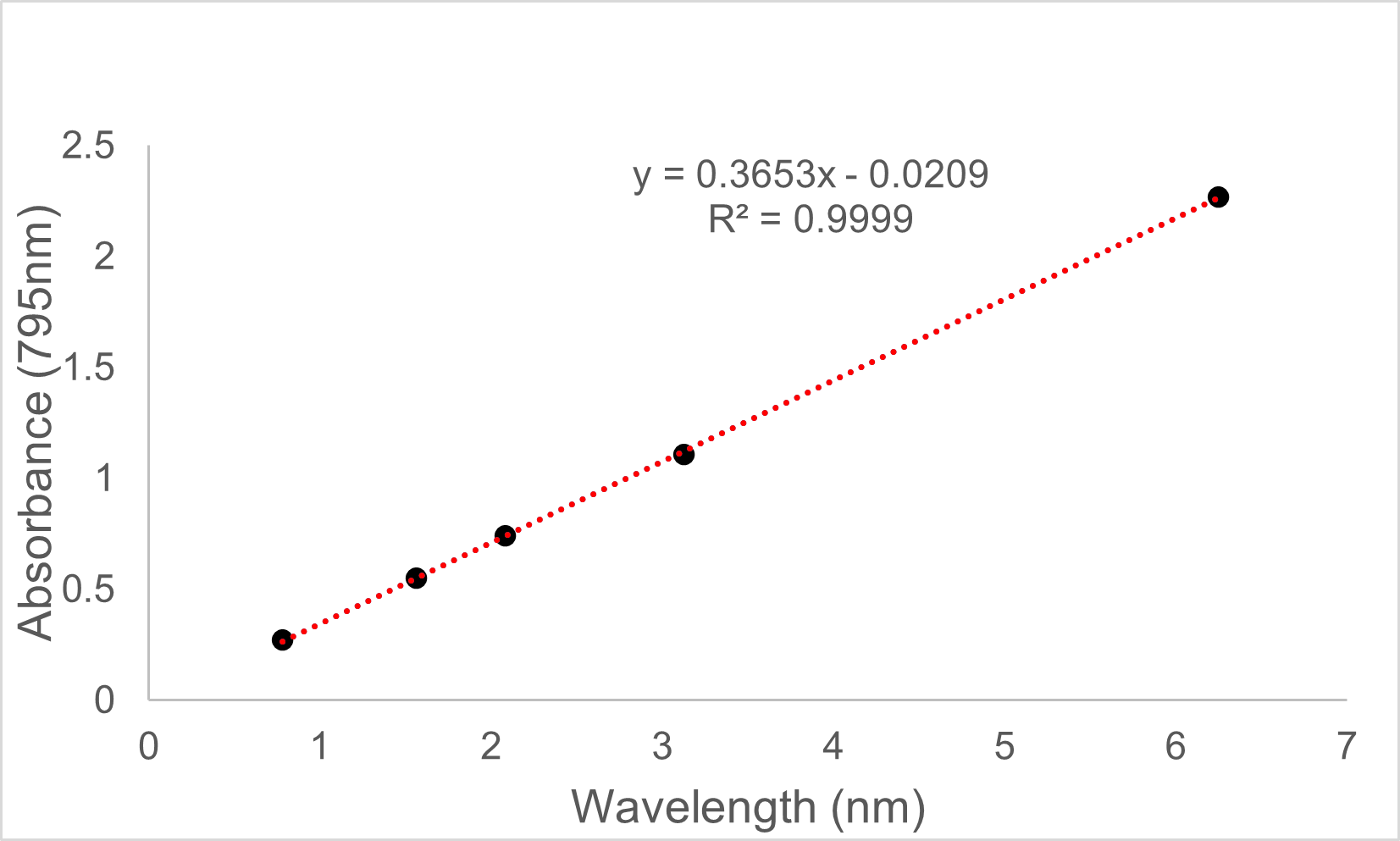


**Figure S1. The standard curve of IR780 was calculated by measuring the absorbance of IR780 with different concentrations at 795nm.**


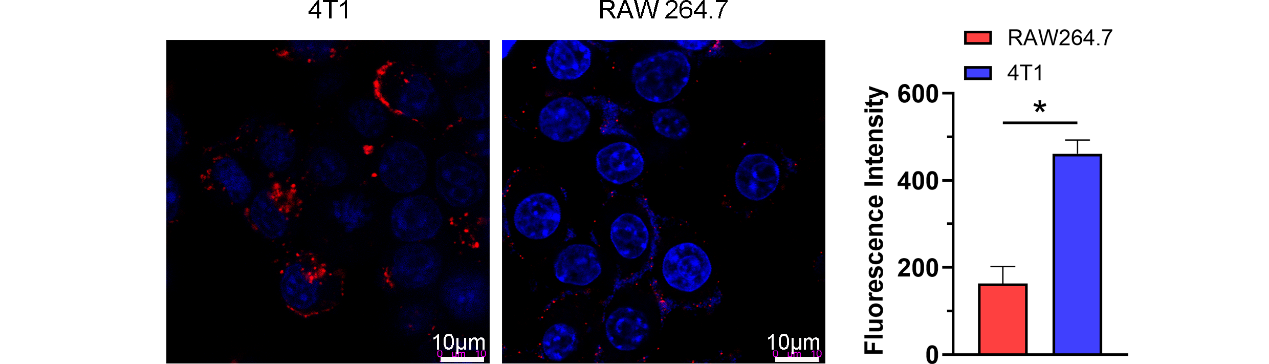


**Figure S2. Evaluation of the uptake efficiency of IR780@PLGA@HM between RAW264.7 cells and 4T1 cells.** Cells’ uptake of nanoparticles at the 4th hour was imaged by CLSM (DiI labeled IR780@PLGA as red; Scale bar =10 μm). Quantification results were presented. Statistical significances were calculated via Student’s t-test, *p < 0.05.


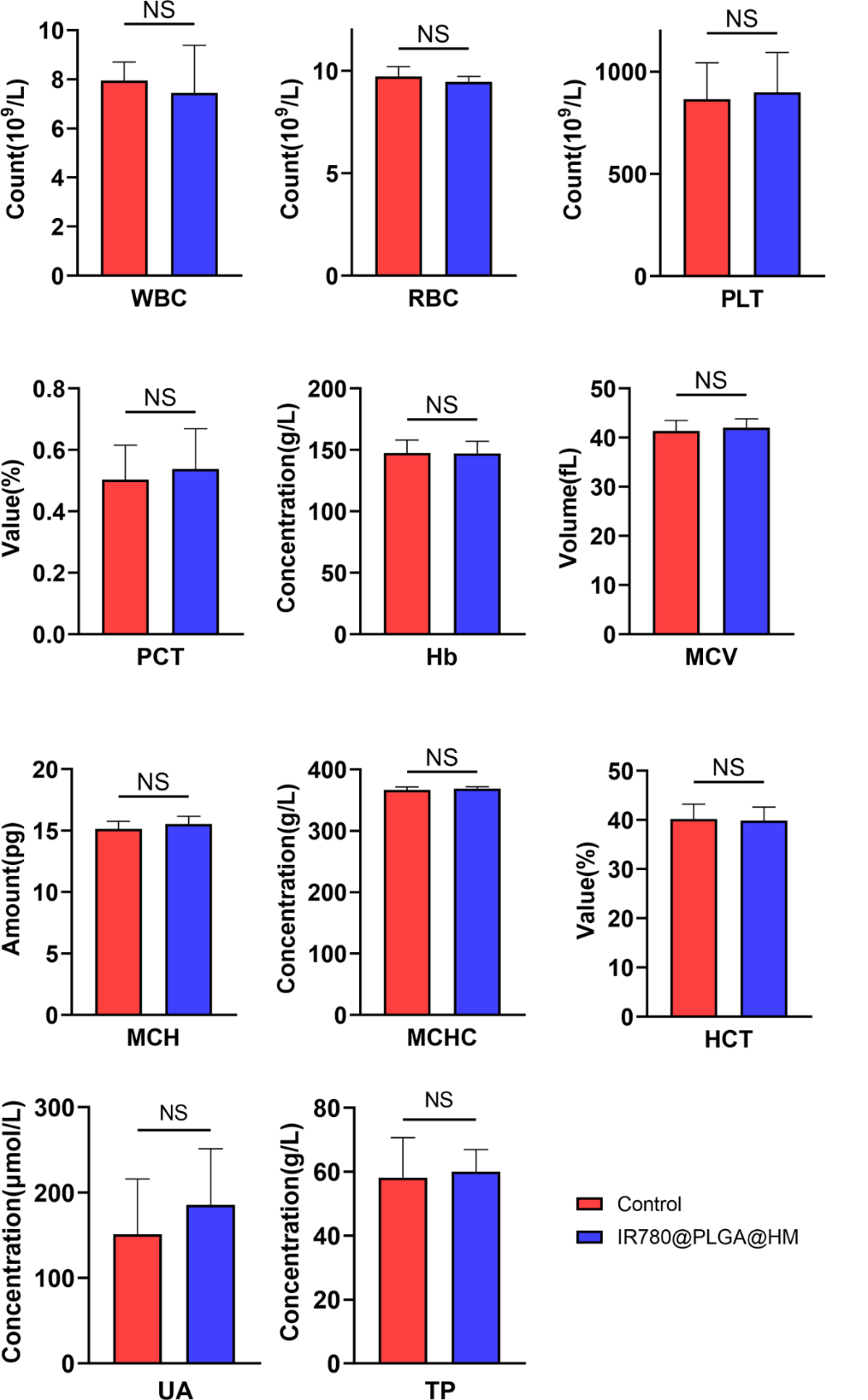


**Figure S3. Evaluation of the influence of IR780@PLGA@HM nanoparticles on blood cell counts, and liver and kidney function after intravenous injection.** White Blood Cell (WBC), Red Blood Cell (RBC), Platelets (PLT), Thrombocytocrit (PCT), Hemoglobin (Hb), Mean Corpuscular Volume (MCV), Mean Corpuscular Hemoglobin (MCH), Mean Corpuscular Hemoglobin Concentration (MCHC), Hematocrit (HCT), Uric acid (UA) and Total protein (TP) were measured. Statistical significances were calculated via Student’s t-test. NS meant no significant difference.


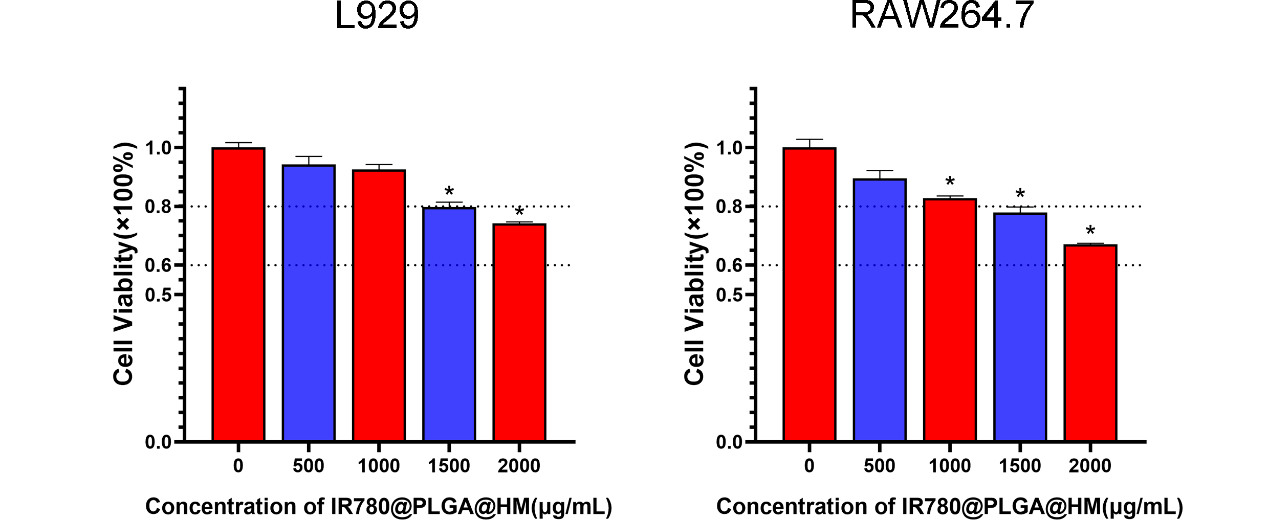


**Figure S4. Evaluation of sonodynamic cytotoxicity of IR780@PLGA@HM nanoparticles.** Cell viability of L929 cells and RAW264.7 cells were evaluated 24 hours after being treated with IR780@PLGA@HM at different concentrations under ultrasound irradiation (1 W/cm2, 1 MHz, 10 seconds on and 10 seconds off for 2 minutes). Statistical significances were calculated via Student’s t-test, *p < 0.05 (compared with the PBS group).


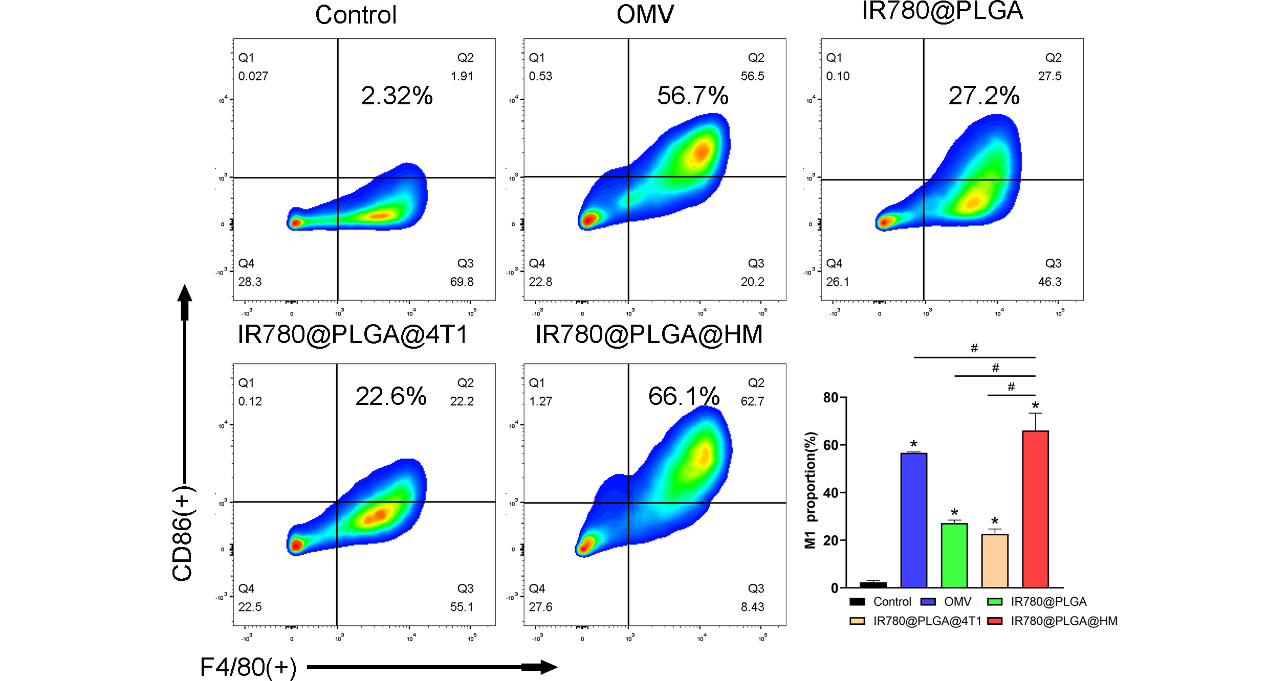


**Figure S5. Evaluation of bone marrow-derived macrophage (BMDM) polarization in vitro.** Flow cytometry showed regulation of nanoparticles on BMDM polarization in vitro, with quantification results. Statistical significances were calculated via Student’s t-test, *p < 0.05 (compared with the PBS group). #p < 0.05 (difference between compared groups).


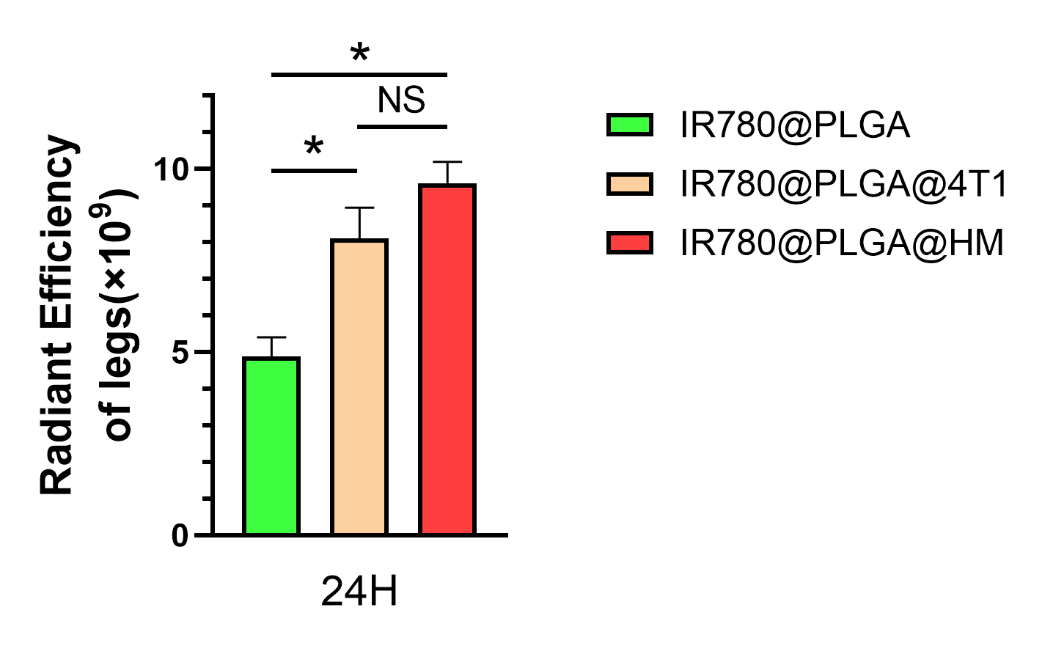


**Figure S6. The fluorescence intensity of legs at 24h was quantified.** Statistical significances were calculated via Student’s t-test, *p < 0.05. NS meant no significant difference.


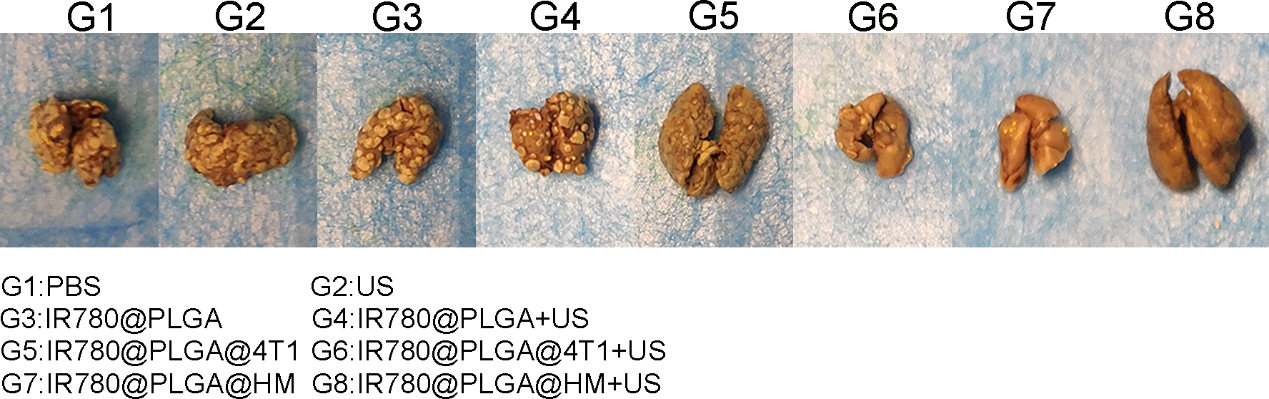


**Figure S7. Tumor metastasis to the lungs was photographed after nanoparticle treatment.**


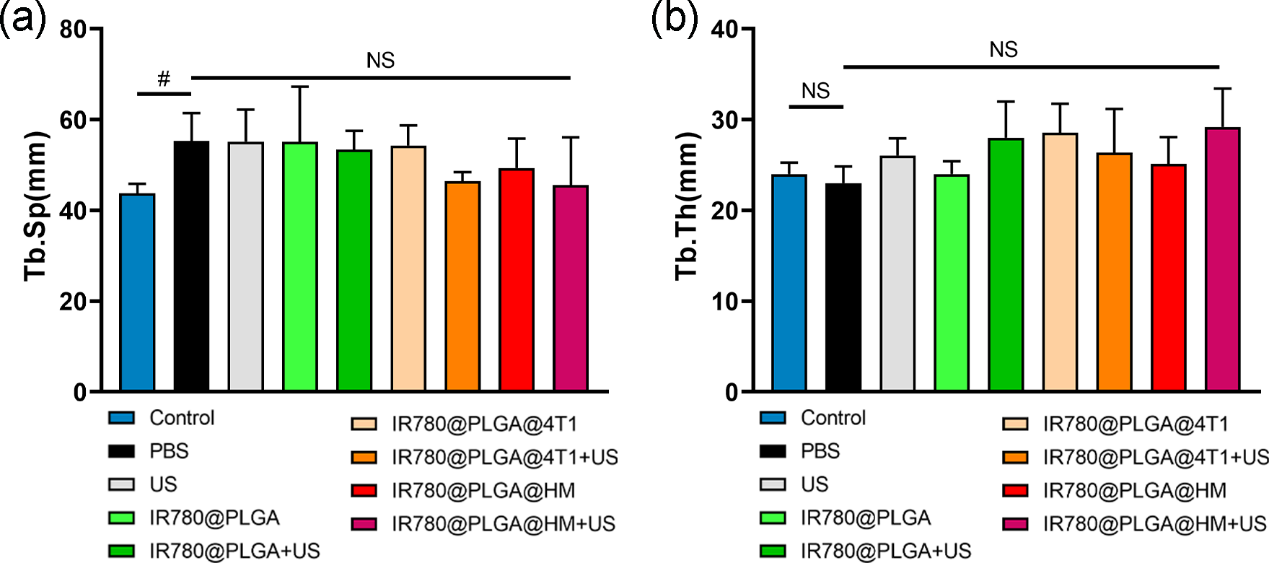


**Figure S8.** **Quantification results of (a) Trabecular separation (Tb. Sp) and (b) Trabecular thickness (Tb. Th) of the tibial plateau area in all groups.** Statistical significances were calculated via Student’s t and One-way ANOVA test, ^#^p < 0.05. NS meant no significant difference.


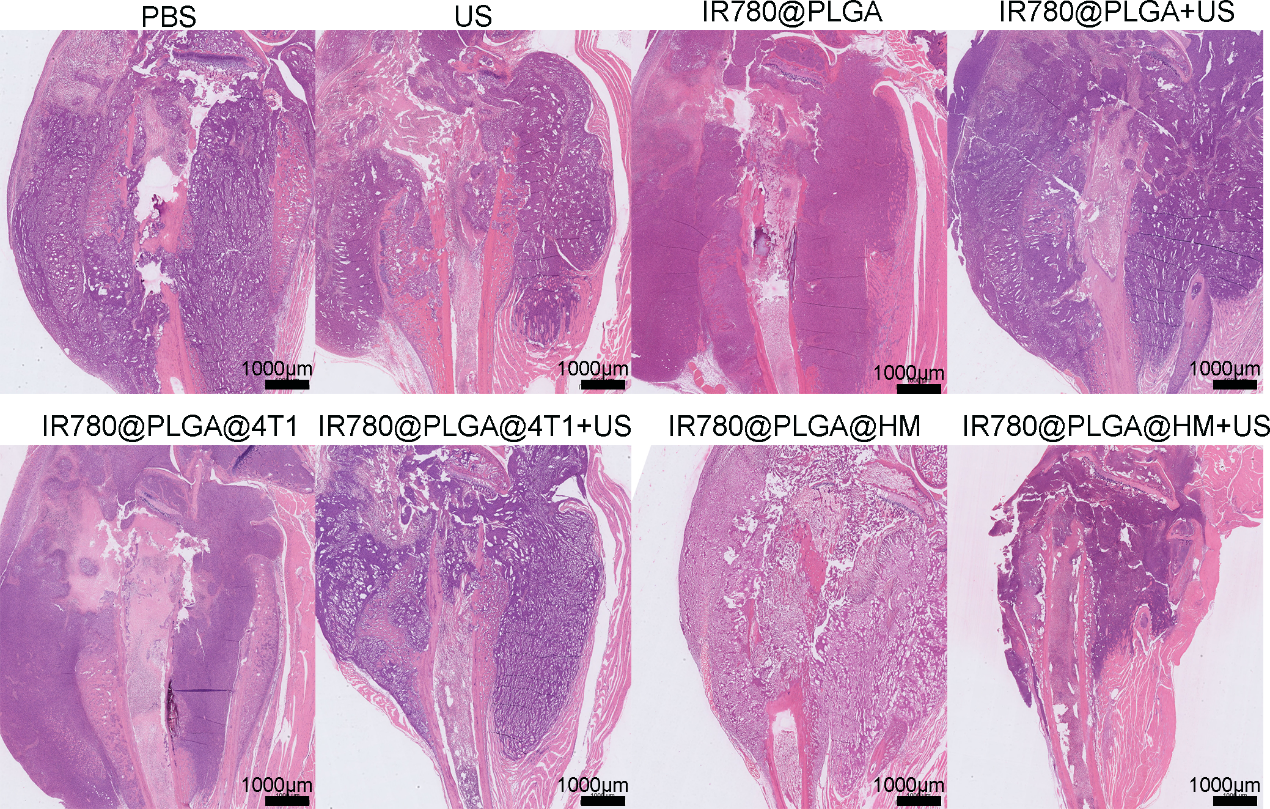


**Figure S9. HE staining of tumor-bearing legs in all groups after nanoparticle treatment.** Scale bar =1000 μm.


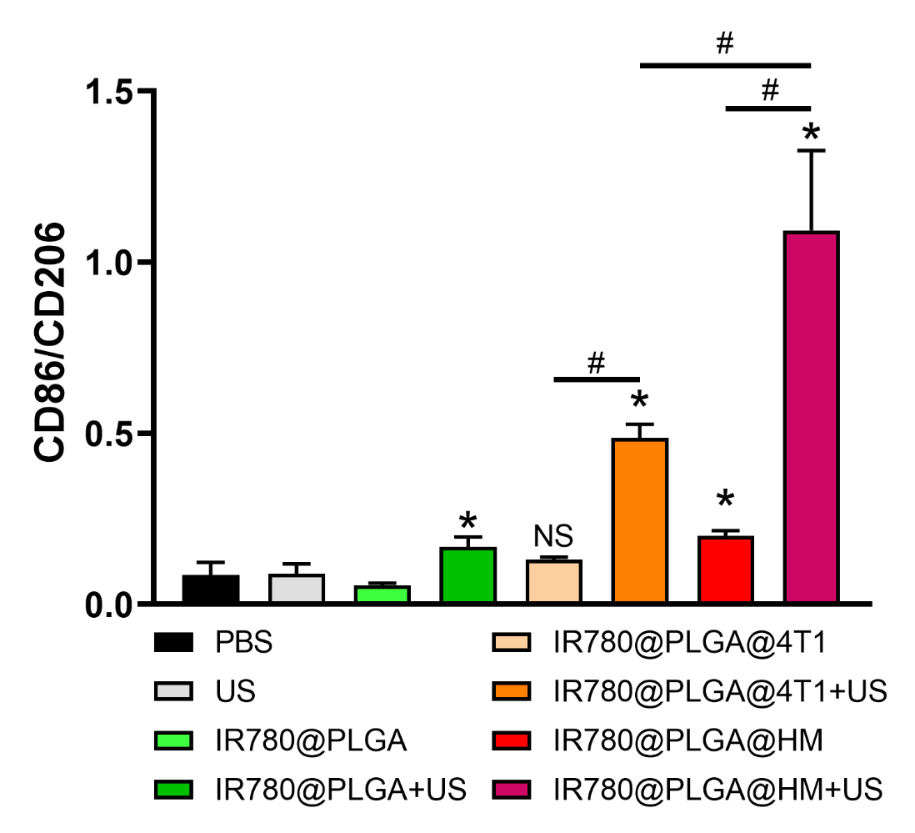


**Figure S10. Quantification results of the CD86/CD206 ratio in all groups of Figure 10A.** Statistical significances were calculated via Student’s t-test, *p < 0.05 (compared with the PBS group). #p < 0.05 (difference between compared groups). NS meant no significant difference.
